# Inter-synaptic learning of combination rules in a cortical network model

**DOI:** 10.3389/fpsyg.2014.00842

**Published:** 2014-08-28

**Authors:** Frédéric Lavigne, Francis Avnaïm, Laurent Dumercy

**Affiliations:** ^1^UMR 7320 CNRS, BCL, Université Nice Sophia AntipolisNice, France; ^2^Université Nice Sophia AntipolisNice, France

**Keywords:** combination rule, cortical networks, dendrites, inter-synaptic learning, priming, synaptic clustering, exclusive OR

## Abstract

Selecting responses in working memory while processing combinations of stimuli depends strongly on their relations stored in long-term memory. However, the learning of XOR-like combinations of stimuli and responses according to complex rules raises the issue of the non-linear separability of the responses within the space of stimuli. One proposed solution is to add neurons that perform a stage of non-linear processing between the stimuli and responses, at the cost of increasing the network size. Based on the non-linear integration of synaptic inputs within dendritic compartments, we propose here an inter-synaptic (IS) learning algorithm that determines the probability of potentiating/depressing each synapse as a function of the co-activity of the other synapses within the same dendrite. The IS learning is effective with random connectivity and without either *a priori* wiring or additional neurons. Our results show that IS learning generates efficacy values that are sufficient for the processing of XOR-like combinations, on the basis of the sole correlational structure of the stimuli and responses. We analyze the types of dendrites involved in terms of the number of synapses from pre-synaptic neurons coding for the stimuli and responses. The synaptic efficacy values obtained show that different dendrites specialize in the detection of different combinations of stimuli. The resulting behavior of the cortical network model is analyzed as a function of inter-synaptic vs. Hebbian learning. Combinatorial priming effects show that the retrospective activity of neurons coding for the stimuli trigger XOR-like combination-selective prospective activity of neurons coding for the expected response. The synergistic effects of inter-synaptic learning and of mixed-coding neurons are simulated. The results show that, although each mechanism is sufficient by itself, their combined effects improve the performance of the network.

## Introduction

The adaptation of behavior to complex environments relies on the ability of the brain to select appropriate actions according to arbitrary combinations of stimuli (Miller, 1999; Bunge et al., 2003; Muhammad et al., 2006). The prefrontal cortex plays a critical role in this process and is an essential structure for the processing of rule-based behavior and response selection (Passingham, 1993; Wise et al., 1996; Hoshi et al., 1998; White and Wise, 1999; Asaad et al., 2000; Murray et al., 2000; Toni et al., 2001; Wallis et al., 2001; Wallis and Miller, 2003; Brasted and Wise, 2004; Genovesio et al., 2005; Buckley et al., 2009; Badre et al., 2010; Walsh and Anderson, 2013). Further, single-neuron recordings have provided us with invaluable information on the dynamics of the activation of neurons coding for stimuli in real time (e.g., Wallis et al., 2001; Muhammad et al., 2006).

Computational modeling of cortical networks sheds light on the processes of activation of items in working memory, corresponding to populations of neurons coding for stimuli to be recalled (Brunel, 1996; Lavigne and Denis, 2001, 2002; Mongillo et al., 2003; Brunel and Lavigne, 2009) or to populations of neurons coding for responses to be selected (Wang, 2002, 2008; Salinas, 2008; Soltani and Wang, 2010). These models have underlined the critical role that synaptic connectivity in long-term memory plays in these phenomena. However, rule-based behavior requires the cerebral cortex to learn responses to complex combinations of stimuli. A paradigmatic example of such combinations in logical analysis is the exclusive OR (XOR; Minsky and Papert, 1969). For example, when normally flying a plane, the pilot must push the control column to descend and apply back pressure to climb. However, in upside-down flying, the pilot must apply back pressure to descend and push to climb. Then, during aerobatics, the pilot constantly faces a XOR like combination rule.

Understanding which associations have to be learned to perform rule-based tasks and how they are embedded within the synaptic matrix relates to non-linearly separable problems that are central for computational models (Amit, 1988; Xing and Andersen, 2000; Loh and Deco, 2005; Rigotti et al., 2010a,b). Up to now, the solution to non-linearly separable problems such as XOR-like rules has been to consider additional neurons that perform a stage of non-linear processing between the stimuli and responses (Rigotti et al., 2010a,b; Bourjaily and Miller, 2011a,b, 2012). However, the solution of adding additional neurons is effective, at the cost of increasing the size of the network.

Here, we propose an inter-synaptic (IS) learning algorithm of rule-based combinations that does not require additional neurons, and show that it can work in synergy with additional neurons. This IS learning algorithm solves the problem of clustering synapses that are combined within the same dendrite, by embedding a recently found property in which the potentiation of synapses that are co-active and co-localized within dendritic branches is amplified (Govindarajan et al., 2011). The proposed algorithm formalizes the reported inter-synaptic amplification of potentiation of nearby synapses within the dendrites, and extends it to include inter-synaptic amplification of depression. Within this framework, we investigate the necessary and sufficient conditions of non-linear dendritic integration (Koch et al., 1983; Mel, 1992, 1993; Polsky et al., 2004) and synaptic clustering (Govindarajan et al., 2006; Chen et al., 2011; Takahashi et al., 2012) for inter-synaptic learning of rule-based combinations of stimuli.

### Processing of non-linearly separable XOR-like combinations

**Figure 1:**
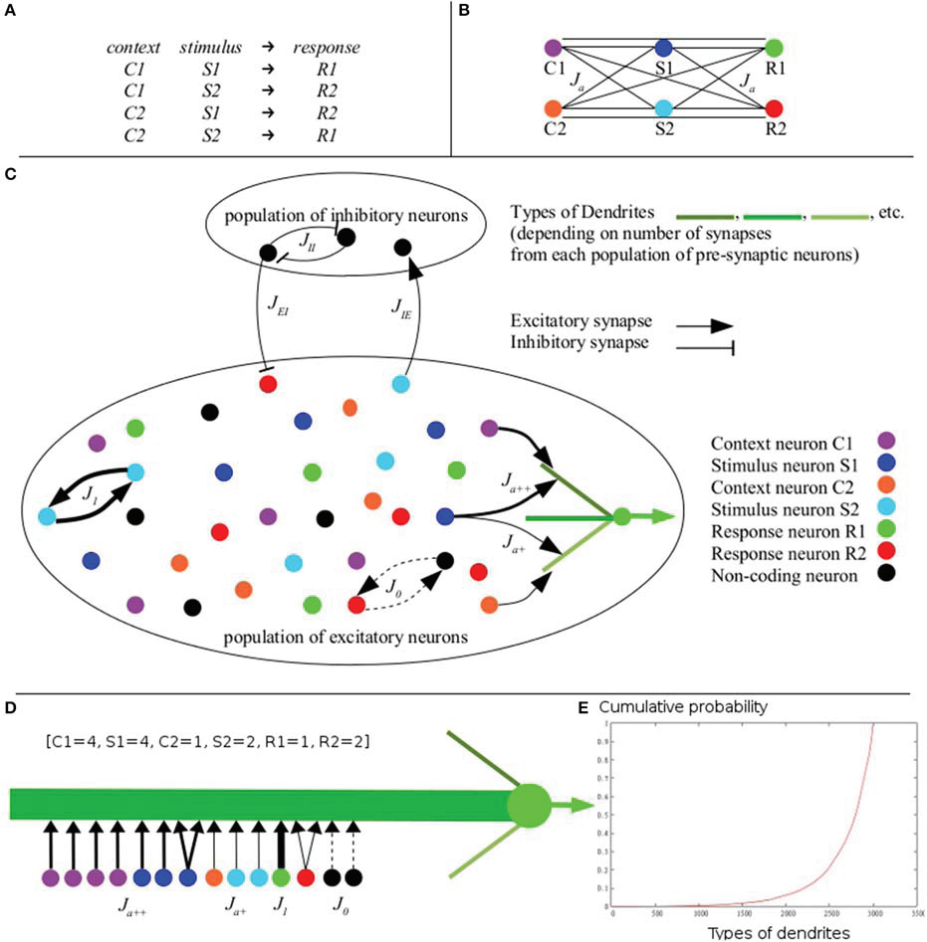
**The XOR-like combination rule. (A)** A typical combination rule requires the subject to give a response to a combination of context and stimulus. Responses (R1 vs. R2) are predicted equally by each individual context (C1 vs. C2) and each individual stimulus (S1 vs. S2), making responses not linearly separable in the space of contexts and stimuli (Considering C1 = C2, S1 = S2, R1 = R2, this combination rule corresponds to a XOR rule). Responses can be discriminated only on the basis of the four combinations of one context and one stimulus. **(B)** Schematic representation of excitatory links between populations of neurons coding for the six items involved in the rule: stimuli S1 (dark blue) and S2 (light blue), contexts C1 (purple) and C2 (orange), and responses R1 (green) and R2 (red). Hebbian learning of the XOR-like rule generates equal efficacy between each context, each stimulus and each response (black lines). **(C)** Architecture and synaptic connectivity of the cortical network model embedding IS learning (for clarity, all connections are not displayed; see Table 1 for values of connectivity). Excitatory neurons are selective for distinct stimuli (same color codes as in **B**). According to the IS learning algorithm, efficacy values (thickness of the arrows) depend on the activity of other synapses within the same dendrite (precise values of the parameters are given in Table 1). Regarding different types of dendrites of a neuron coding for R1 (green), potentiation is weak with a neuron coding for S1 in the lower dendrite having others contacts with C2 (C2, S1 and R1 are not combined), while it is amplified in the upper dendrite having other contacts with C1 (C1, S1 and R1 are combined). **(D)** Example of a dendrite of type [C1 = 4, S1 = 4, C2 = 1, S2 = 2, R1 = 1, R2 = 2] defined by the number of contacts with pre-synaptic neurons coding for the different contexts, stimuli and responses. This dendrites has 2 synapses from non-single-item (NSI) coding neurons (black). **(E)** Cumulative probability of the different types of dendrites. In the model, the probability of each type of dendrite was calculated exactly according to equations 2 and 3. These probabilities equal—up to the 4th decimal—the ones computed from 500 million simulations of the connectivity within the dendrites via random numbers generation. The sum of all exactly computed probabilities is 1, as we would expect if the probability law is correct (example of 3003 types of dendrites with 8 synapses; *N_E_* = 4000; *f* = 0.1; *N_p_* = 400; *g* = 6; *N_es_* = 8; *N_d_* = 100).

The common denominator of many contextual rules is described by XOR-like combination rules, according to which a given stimulus can predict different responses depending on the context (Figure 1A). As a consequence, responses cannot be selected based on any single stimulus, but rather only based on their combinations. Responses therefore are not linearly separable within the space of stimuli. For example, given a XOR-like rule of context-stimulus-response taken within two contexts, with two stimuli and two responses, learning of the combinations described by XOR-like rule with equal probabilities results in a non-linearly separable problem (Figure 1A; see also **Figure 4E** for simulations and a geometrical representation of the problem).

Non-linearly separable problems have been addressed using multilayer connectionist networks including a hidden layer of neurons (e.g., Rumelhart and McClelland, 1986). Hidden neurons provide the network with an additional level of non-linear processing between neurons coding for the stimuli and neurons coding for the responses. Since then, studies in behaving non-human primates have provided essential information on neuronal activity during the processing of multi-conditional deductive rules (Naya et al., 1996; Wallis et al., 2001; Wallis and Miller, 2003; Muhammad et al., 2006). In addition, studies have reported that mixed-coding neurons—widely distributed over the prefrontal cortex—exhibit elevated activity in response to abstract combinations of stimuli, although without being selective for any particular stimulus or response (Bongard and Nieder, 2010; Rigotti et al., 2013). Those neurons are active in behaving monkeys responding to XOR-like combinations (Wallis et al., 2001; Wallis and Miller, 2003). The spike rates of prefrontal neurons coding for a given response (holding vs. releasing a lever) depended on the combination of match/no-match between two successive image stimuli and of a preceding cue. The XOR component of the rule was assessed by the learning protocol, in which equal probabilities of the combinations of stimuli and responses were ensured during the learning stage. The results showed that, in addition to the activity of coding neurons which was predicted by a single stimulus, cue, or response, the activity of mixed-coding neurons was predicted neither by the stimuli nor the cues alone, but rather only by their combination. Their potential functional role as hidden neurons has led modelers to investigate the inclusion of stimulus-pair selective neurons in the learning of XOR-like combinations (Rigotti et al., 2010a,b; Bourjaily and Miller, 2011a,b, 2012).

Regarding learning at the neuronal level, it is known that neurons coding for different stimuli can be close together and inter-mixed in the same prefrontal area (Miller et al., 1996; Wallis and Miller, 2003). Even more locally, axons of cortical neurons form direct appositions with dendrites of almost all their surrounding neurons, without any preference for any particular neurons (Kalisman et al., 2005; Le Bé and Markram, 2006). This property of random connectivity is consistent with the notion that functional circuits are primarily shaped through the modification of synaptic connections between neurons (Engert and Bonhoeffer, 1999; Maletic-Savatic et al., 1999; Lendvai et al., 2000; Yuste and Bonhoeffer, 2004). Computational modeling of synaptic learning has investigated how synaptic matrices can be obtained by Hebbian learning of stimuli in an initially unstructured network of randomly connected neurons (Brunel, 1996; Brunel et al., 1998; Mongillo et al., 2003). However, the random connectivity between neurons and the absence of *a priori* wiring of the network constrain Hebbian learning of XOR combinations based solely on neurons coding for individual stimuli and responses. Indeed, the Hebb rule locally updates values of synaptic efficacy as a function of only the pre- and post-synaptic neuronal activities (Hebb, 1949; Bliss and Lomo, 1973; Bliss and Collingridge, 1993; Kirkwood and Bear, 1994). In the case of XOR-like combinations of context-stimulus-response taken within two contexts, two stimuli and two responses (Figure 1A), the equal probabilities of the various triadic combinations result in equal probabilities for the pairwise combinations of each context with each stimulus, each stimulus with each response, and each context with each response (Figures 1B). A consequence of this is that local Hebbian learning based on the average activities of pre- and post-synaptic neurons generates the same efficacy values for synapses connecting pairs of neurons coding for the contexts, stimuli and responses (see Rigotti et al., 2010a,b; Fusi et al., 2007: Bourjaily and Miller, 2011a,b, 2012 for discussions). Such synaptic matrices thus do not allow the network to activate different responses for different combinations of context and stimulus.

Recent models of the cerebral cortex have demonstrated a critical role for mixed-coding neurons observed in experiments. In these models, learning has been addressed in networks embedding neurons coding for individual stimuli and responses alongside mixed-coding neurons responding to combinations of stimuli (Rigotti et al., 2010a,b, 2013; Bourjaily and Miller, 2011a,b, 2012). However, contrary to the hidden neurons of connectionist networks, mixed-coding neurons of cortical network models are not *a priori* wired and have been proved sufficient to perform XOR-like rules. Indeed, these neurons provide the network with an additional stage of non-linear processing, in line with that of the hidden units in multilayer connectionist networks. However, this solution for the learning and processing of XOR-like combinations requires additional neurons, while other candidate mechanisms could also be envisaged without the need for additional neurons, and could even improve the function of mixed-coding neurons.

### Non-linear dendritic integration

A growing field of research points to dendritic non-linear integration of synaptic inputs as a mechanism that could contribute to the processing of XOR-like combinations at the level of neurons. Electrophysiological experiments have shown that non-linear integration occurs not only in the cell body but also at an earlier stage within the dendritic arbor (Koch et al., 1983; Johnston et al., 1996; Magee et al., 1998; Hausser et al., 2000; London and Hausser, 2005; Sjöström et al., 2008; Spruston, 2008; Stuart et al., 2008; Larkum et al., 2009; Lavzin et al., 2012; Major et al., 2013). Experiments have also shown that synaptic inputs from nearby sources are non-linearly summed (Koch et al., 1983; Tuckwell, 1986; Schwindt and Crill, 1995; Polsky et al., 2004), whereas inputs from distant dendritic branches are linearly summed (Poirazi et al., 2003a,b; Gasparini et al., 2004; Polsky et al., 2004; Gasparini and Magee, 2006; Losonczy and Magee, 2006; Silver, 2010). The possibility of obtaining non-linear integration as a function of synapse co-localization in dendrites allows pyramidal neurons to multiply incoming signals at the dendritic stage before summing them at the somatic stage (Koch et al., 1983; Rall and Segev, 1987; Shepherd and Brayton, 1987; Mel, 1992, 1993, 2008; Sidiropoulou et al., 2006; Cazé et al., 2013). Such Σ Π-neurons compute weighted products in addition to weighted sums, extending their range of computational operations (Durbin and Rumelhart, 1989; Poirazi and Mel, 2001; Poirazi et al., 2003a,b; Polsky et al., 2004). Here the dendritic tree is a computational unit (see Branco and Hausser, 2010) that plays the role of the hidden layer of a multilayer network (Schiller et al., 2000; Chiarello et al., 2003; Silver, 2010). Embedded at the single-neuron scale, this computational property is an important feature of the modulatory influences that affect sensory processing (Salinas and Abbott, 1995, 1997; Pouget and Sejnowski, 1997; Deneve and Pouget, 2003; Salinas, 2004). Further, with a sufficient number of dendrites, a neuron can compute all positive non-linearly separable Boolean functions (Cazé et al., 2013). This makes neurons with non-linear dendritic integration good candidates for performing combinations of synaptic inputs involved in XOR-like rules.

**Figure 2:**
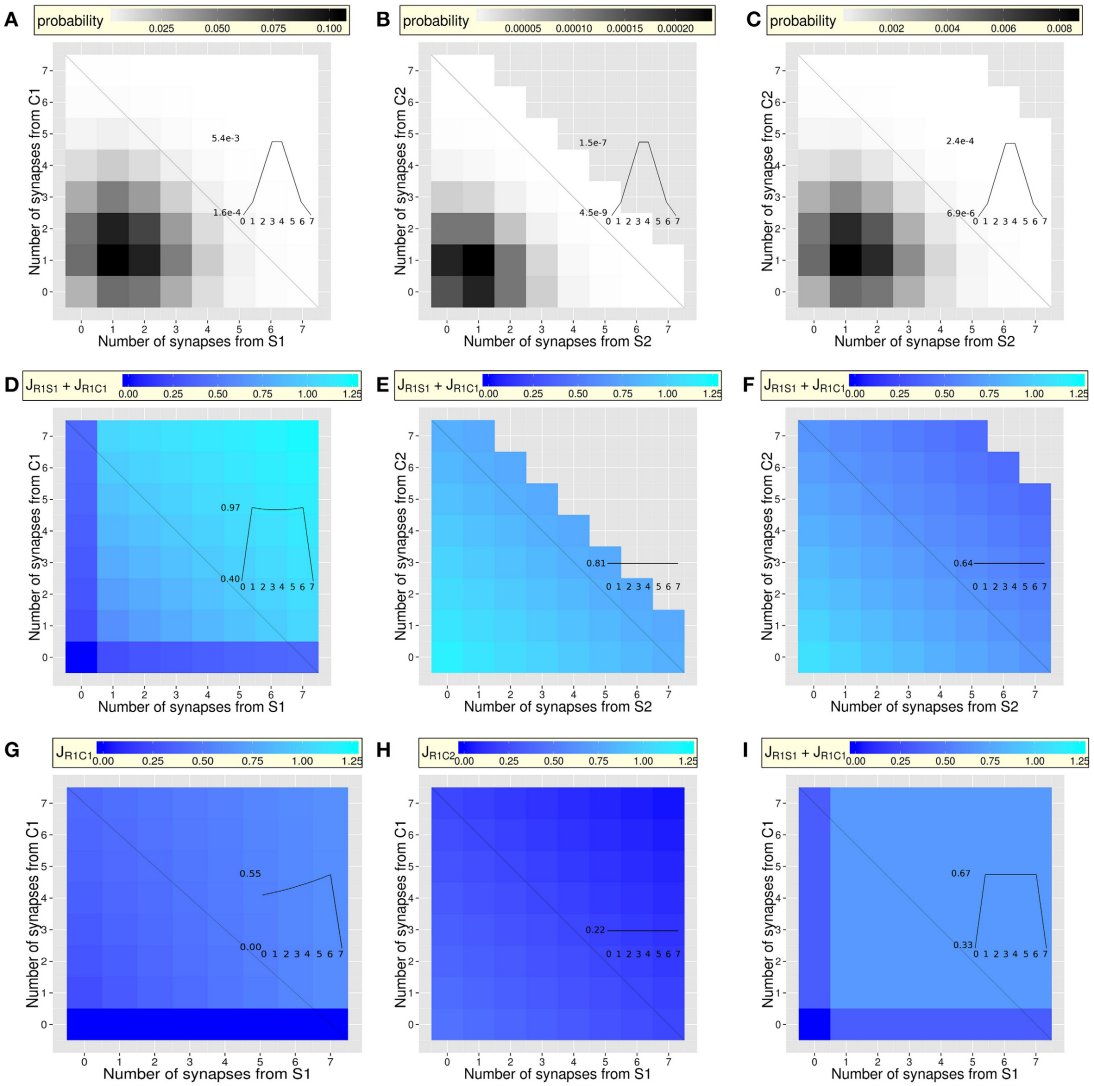
**Probability of the types of dendrites (A–C) and synaptic efficacy after inter-synaptic learning (D–I), as a function of the number of synapses from neurons coding for the context and neurons coding for the stimulus (case of dendrites of neurons coding for response R1)**. For clarity, synapses from NSI-coding neurons are not reported (see Figure 1D for details). Insets focus on the probability or efficacy (y-axe) along the anti-diagonal of each figure, where the number of synapses from the combined items is constant (seven synapses). The x-axe (black anti-diagonal lines) corresponds to the number of synapses from the Stimulus (the inverse number of synapses from the Context). The effects of the increasing number of co-active synapses (from the context and from the stimulus) appear along the diagonal, while the effects of the increasing (decreasing) number of synapses from the stimulus (context) appear on the shape of the curve along the anti-diagonal (insets). Non-flat shapes reveal the effects of IS learning. **(A–C)** After random connectivity: heterogeneous distribution of the probability of the types of dendrite, defined by the numbers of synapses from pre-synaptic neurons coding for contexts (C1 or C2), stimuli (S1 or S2) and responses (R1 and R2): [C1, S1, C2, S2, R1, R2]. To better display slight variations along the scale, only dendrites occurring with a probability higher than 1.28 × 10^−9^ are displayed (note the different scales between graphs **A**, **B** and **C**). For a given dendrite, this corresponds to a maximum of seven synapses from pre-synaptic neurons coding for C1 and/or S1. **(A)** Overall probability (over all synapses from C2, S2, R1, R2, and NSI-coding neurons) of the types of dendrites, as a function of the number of synapses from neurons coding for C1 and S1 (mean = 0.02; min = 2.80 × 10^−9^; max = 0.11). **(B,C)** Two types of relevant dendrites (see text). **(B)** Overall probability of [C1 = 4, S1 = 4, C2, S2, R1, R2] dendrites (averaged over all types) with any number of synapses from C2, S2, R1, and R, as a function of the number of synapses from neurons coding for C2 and S2 (mean = 3.52 × 10^−5^; min = 7.50 × 10^−10^; max = 2.35 × 10^−4^). **(C)** Same as **(B)** but regarding [C1 = 2, S1 = 2, C2, S2, R1, R2] dendrites (mean = 0.001; min = 9.10 × 10^−10^; max = 8.65 × 10^−3^). (**D-F**) Amplification of potentiation and depression through IS learning of the combination C1S1R1: Sum of the efficacy of two synapses *J*_*R*1−*S*_ + *J*_*R*1−*C*_ from pre-synaptic neurons coding for the stimulus and the context for the different types of dendrites defined in **(A–C)**. Amplification of potentiation and depression with the number of synapses in the dendrites indicates that IS learning of the C1S1R1 combination generates different efficacy values in different dendrite. **(D)** Amplification of the potentiation along the diagonal when the number of synapses increases (mean = 0.83, min = 0 and max = 1.22, compare to **I**). Synaptic efficacy is weak when one type of synapses (from C1 or S1) is absent along the anti-diagonal (inset). **(E,F)** Two types of dendrites having [4C1, 4S1] or [2C1, 2S1] synapses further show the efficacy of two synapses *J*_*R*1−*S*1_ + *J*_*R*1−*C*1_ as a function of the number of synapses from C2 and S2. Overall amplification of potentiation is stronger in [C1 = 4, S1 = 4, C2, S2, R1, R2] dendrites (**E;** mean = 0.89; min = 0.76 max = 1.17) than in [C1 = 2, S1 = 2, C2, S2, R1, R2] dendrites (**F;** mean = 0.67; min = 0.42; max = 1.08). In both cases, amplification of depression increases with the number of synapses from C2 and S2, also learned in combination with R1 but generating depression of the C1R1 and S1R1 synapses when learning a C2S2R1 combination. **(G,H)** Efficacy *J*_*R*1*C*1_ of synapses from C1 learned in combination with S1 and R1 (learned context, **G**) or of *J*_*R*1*C*2_ of synapses from C2 not learned in combination with S1 and R1 (context change, **H**). Overall synaptic efficacy from neurons coding for the context is stronger when the context was learned in the same combination (C1, **G;** mean = 0.41; min = 0; max = 0.61) than when it was not (C2, **H;** mean = 0.27; min = 0; max = 0.44). (**I**) Classical Hebbian learning of the combination C1S1R1: As in **(D)**: synaptic efficacy is strong (mean = 0.58). However, the efficacy does not depend on the number of synapses in the dendrite along the diagonal, due to the absence of inter-synaptic amplification of potentiation (min = max = 0.67). The efficacy is weak when one type of synapses (from C1 or S1 or both) is absent along the anti-diagonal (inset).

Considering the role of non-linear dendritic integration, the problem that must be solved is that the computation of combinations of specific contexts, stimuli, and responses according to XOR-like rules are not known *a priori* and must be learned. The synaptic inputs to be combined must be amplified by non-linear integration, while inputs that will not be combined must not be amplified. The central question then pertains to learning in dendrites: how are the synaptic inputs that will be non-linearly integrated grouped within the same dendrite, while those that will be linearly summed are in separate dendrites? Hebbian learning generates identical values for the efficacy of synapses that connect neurons with equivalent average activities of pre- and post-synaptic neurons (Brunel et al., 1998; Figures 1B, 2I). When combined with the random distribution of synapses in the dendritic branches, non-linear integration would thus equally amplify all possible combinations of synapses within the dendrites. Hebbian learning would therefore not allow dendrites to discriminate between learned and non-learned combinations. Instead, such discrimination would require that, given an initial random allocation of synapses within the dendrites, the clustering of synapses to be combined within the same dendrite would thus rely on an increased synaptic efficacy of these synapses. How the functional efficacy values are learned is still an open question.

### Synaptic clustering

Modeling approaches have investigated the integration of synaptic inputs as a function of their combinations with other inputs (Dehaene et al., 1987; Dehaene and Changeux, 1989; Kühn et al., 1989; Baird, 1990; Phillips et al., 1995; Phillips and Singer, 1997; Kay et al., 1998; Körding and König, 2000a). For example, in a biologically inspired model of the cerebral cortex, a non-Hebbian learning algorithm updated synaptic efficacy at pairs of functionally dependent synapses as a function of the activity of the post-synaptic neuron and of two pre-synaptic neurons (Körding and König, 2000a,b, 2001a,b). This network can learn XOR-like combinations, under the assumption of *a priori* wiring of layers as a function of the items they code for in memory (contexts, types of stimuli, responses (see Körding and König, 2000a). This would not be possible in the opposite case, i.e., where there is random connectivity. In that case, a post-synaptic neuron (e.g., coding for a response R1) with randomly distributed synaptic contacts would receive all possible pairs of linked synapses (from C1S1, C1S2, C2S1, C2S2), and as a consequence non-Hebbian learning would increase potentiation at all linked synapses corresponding to learned combinations (C1S1R1, C2S2R1). Equal numbers of pairs of synapses would benefit from increased potentiation. As a result, the total value of synaptic efficacy from each context or stimulus taken alone would be equal, meaning that the network would not be able to discriminate between learned and non-learned combinations. This dependency of learning on the wiring of the network is problematic for our understanding of the learning of XOR-like combinations without *a priori* functional link between synapses as a function of what the neurons code for.

Neurophysiological studies on synaptic clustering have provided us with invaluable information on the functional links that exist between synapses during learning (Govindarajan et al., 2006; Larkum and Nevian, 2008; Larkum et al., 2009). Synaptic connections between neurons rely strongly on dendritic spines, where post-synaptic signaling is generated (Segal, 2005; Harms and Dunaevsky, 2007). Dendritic spines are spatially clustered at the level of individual dendrites (De Roo et al., 2008; Fu et al., 2012), and synaptic clusters are widely distributed on apical and oblique branches of pyramidal neurons (Yadav et al., 2012). During learning, dendritic spines emerge in clusters (Fu et al., 2012), suggesting that clustering depends on learning and can persist after training (Yadav et al., 2012). In accordance with the clustered plasticity hypothesis (Govindarajan et al., 2006), one consequence of learning on synaptic clusters is that synapses within the same cluster are more likely to transmit the same information than synapses dispersed throughout the dendritic arbor (Chen et al., 2011; Takahashi et al., 2012). As a result of this, not only could clusters improve non-linear integration locally within a given dendrite (Poirazi and Mel, 2001; Poirazi et al., 2003a,b), but clustered synapses are also likely to strengthen contacts with functionally related pre-synaptic neurons. Different computations can then take place simultaneously within different dendrites of a single neuron (Polsky et al., 2004; Gasparini and Magee, 2006; Rabinowitch and Segev, 2006a,b).

Simulations of learning in a compartmental model of a neuron can generate synaptic efficacy mosaics, i.e., spatially segregated clusters within which a group of synapses transmitting correlated inputs dominates other groups and exhibits locally stable potentiation (Iannella et al., 2010). A recent experimental study reported that, during learning, spine head size—which is a good approximation of synaptic strength—increases more within clusters than in isolated spines (Fu et al., 2012). In addition, another recent set of experiments has shown that the simultaneous pseudo-synchronous stimulation of two synapses leads to a total efficacy that is stronger when the synapses are on the same dendrite than when they are on different dendrites (Govindarajan et al., 2011). These results open the way toward the mathematical formalism of learning within dendrites as a function of combinations of multiple synaptic inputs.

## Methods

We describe here the architecture of a cortical network of excitatory neurons that is regulated by inhibitory feedback. In this network, items (contexts, stimuli, and responses) are coded by populations of excitatory neurons. These coding neurons exhibit an activity that is selective for each item presented individually. The populations of coding neurons are denoted by *P*_1_, …, *P_g_*, not referring to a specific context, stimulus or response (Methods—Network Architecture). Other coding neurons do not exhibit any activity that is selective for any single item. We will consider later a fraction of these neurons that respond to combinations of items (mixed-coding neurons; results Section Synergistic Effects of IS Learning and of Mixed-coding Neurons). Each neuron has a constant number of dendrites, each of them having a constant number of synapses. Synaptic connections between pre-synaptic neurons and the dendrites of post-synaptic neurons are random (Methods—Synaptic Connectivity). This new architecture makes it possible to propose a new inter-synaptic (IS) learning algorithm that takes into account not only the activity of the pre- and post- synaptic neurons, but also the other active synapses within the same dendrite. The main idea is that, in each individual dendrite, the potentiation or depression of a given synapse between a post-synaptic and a pre-synaptic neuron is amplified as a function of the number of other synapses that are in contact with other active pre-synaptic neurons (Methods—Inter-synaptic Learning). The IS learning algorithm allows the learning and processing of XOR-like combinations when non-linearity is introduced into the current dynamics of NMDA synapses (Methods—Dendritic and Neuronal Dynamics. Equation 22; Results—Selectivity and Responsiveness of the Different Types of Dendrites).

### Network architecture

Our model includes a biophysically realistic cortical network of *N_E_* excitatory pyramidal cells whose activity is regulated by *N_I_* = 0.25 *N_E_* inhibitory inter-neurons (Abeles, 1991; Braitenberg and Schütz, 1991), with a probability of C = 0.2 of having a synapse from any pre-synaptic neuron to any post-synaptic neuron (Figure 1C). *g* populations of coding excitatory neurons, called *P*_1_, …, *P_g_*, encode *g* items (either contexts, stimuli or responses), and 40% of the excitatory neurons do not encode any particular single item. Each population *P* of coding neurons corresponds to a low fraction *f* << 1 of the *N_E_* excitatory neurons. Excitatory and inhibitory neurons receive external noise from other cortical areas, obeying a Poisson process of rate ν_ext_ = 15 Hz, leading to average values for activities of 3 Hz for excitatory neurons and 9 Hz for inhibitory interneurons (Burns and Webb, 1976; Koch and Fuster, 1989). The negative retroaction by inhibitory interneurons prevents the runaway propagation of activation and regulates population dynamics in the network.

### Synaptic connectivity

Neurons in the network are connected through four types of synapses. Synaptic efficacies between excitatory neurons (EE) are subject to variations due to learning. In contrast, synaptic efficacies involving inhibitory neurons, i.e., excitatory to inhibitory (IE), inhibitory to excitatory (EI), or inhibitory to inhibitory (II), are not subject to learning. We first analyze here network connectivity at the scale of neurons and at the scale of dendrites.

Every post synaptic neuron *i* has a probability *C* of having a synapse with a pre-synaptic neuron. We consider that neurons do not self-connect and that each neuron has exactly the average number of pre-synaptic contacts *C*(*N_E_* + *N_I_* − 1). The *N_E_* excitatory neurons are grouped into *g* populations of *fN_E_* neurons, *P*_1_, …, *P_g_*, coding for items in memory (*f* is the coding level, or fraction of neurons coding for a given item in a population), and the remaining excitatory neurons that are not selective for any single item (not in any population) are called non-single-item (NSI) coding neurons. Every inhibitory inter-neuron is connected to *CfN_E_* neurons of *P*_1_, …, *P_g_*, to *CN_E_*(1 – gf) NSI coding neurons and to *C*(*N_I_* − 1) inhibitory inter-neurons. Every excitatory post-synaptic neuron *i* has a set of *N_d_* dendrites. A dendrite has *N_es_* excitatory (and *N_is_* inhibitory) synapses connecting the post-synaptic neuron *i* to pre-synaptic neurons from the populations *P*1, …, *Pg*, and to pre-synaptic NSI-coding neurons (and to pre-synaptic neurons from the pool of inhibitory inter-neurons). Therefore, an excitatory neuron has a total number of excitatory synapses *N_d_N_es_* = *C*(*N_E_* − 1) and a total number of inhibitory synapses *N_d_N_is_* = *CN_I_*.

Dendrites are defined by their type, denoted [*n*_1_, *n*_2_, …, *n_g_*], with *n*_1_ representing the number of synapses from pre-synaptic neurons of population *P*_1_, …, and *n_g_* representing the number of synapses from pre-synaptic neurons of population *Pg*, with *n*_1_ + … + *n_g_* ≤ *N_es_*. The *N_es_* − (*n*_1_ + … + *n_g_*) other excitatory synapses are from NSI-coding pre-synaptic neurons, and *N_is_* inhibitory synapses are from inhibitory inter-neurons. We note that synapses are not located with a specific order within a dendrite. An example of a dendrite is shown in Figure 1D (belonging to a post-synaptic neuron of the population coding for *R1*). Here *g* = 6 (two contexts, two stimuli and two responses) and the populations *P*_1_, …, *P*_6_ are respectively called C1 (context1), S1 (stimulus1), C2 (context2), S2 (stimulus2), R1 (response1) and R2 (response2). The type of the dendrite is *[4, 4, 1, 2, 1, 2]*. As *N_es_* = 16 there are 2 remaining synapses from NSI-coding presynaptic neurons. For clarity, the *N_is_* = 4 inhibitory synapses are not displayed.

Connections between post-synaptic and pre-synaptic neurons are random. This generates a set of dendrite types, each having its own probability of occurrence. Random synaptic connectivity corresponds to a random allocation of every pre-synaptic neuron with every dendrite of every post-synaptic neuron. To do this exactly, we describe the probabilistic law of allocation of pre-synaptic neurons to dendrites and then allocate dendrites to each neuron according to that law. This has two advantages. First, we can mathematically study the dendrite's distribution in the network, and second we can guarantee the probabilities of occurrence of the different types of dendrites and overcome the problem of dealing with small populations.

In order to compute the probability of occurrence of a dendrite of type T, one has to first compute the total number N of ways to connect a post-synaptic neuron to pre-synaptic neurons through synapses within the dendrite. The desired probability is then simply the number of ways of connecting a post-synaptic neuron to pre-synaptic neurons through a dendrite of type T divided by *N*. This is what we investigate below.

The number of ways of connecting a post-synaptic neuron with *n* pre-synaptic neurons through *k* synapses is $\Gamma_{n}^{k}=C_{n+k-1}^{k}=\frac{(n+k-1)!}{k!(n-1)!}$. This corresponds to the number of non-ordered words (synapses are not located with a specific order within a dendrite) of size *k*, allowing repetitions, made from an alphabet of size *n*. For example if *k* = 2, *n* = 4 and if we call A, B, C, D the 4 pre-synaptic neurons, then there is Γ^2^_4_ = 10 possibilities which are AA, BB, CC, DD, AB (= BA), AC (= CA), AD (= DA), BC (= CB), BD (= DB), CD (= DC).

Using that result, we can now compute the probability that a post-synaptic neuron *i*, from any population *P_v_* (1 ≤ *v* ≤ *g*), has a dendrite of type [*n*_1_, *n*_2_, …, *n_g_*]. Given that neurons do not self-connect, the total number of ways of connecting *N_E_* − 1 excitatory neurons to a post-synaptic neuron *i* through a dendrite of *N_es_* synapses is Γ^*N*_*es*_^_*N*_*E*_ − 1_.

The question then becomes: in how many ways can the *N_E_* − 1 excitatory neurons be connected to neuron *i* of population *P_v_* through the *N_es_* synapses of a dendrite of type [*n*_1_, *n*_2_, …, *n_g_*]?

For the sake of simplicity, we can first set *v* to 1, meaning that neuron *i* is taken in population *P*_1_. We note that *N_p_* = *fN_E_* the number of neurons in each population of coding neurons. There are Γ^*n*_1_^_*N*_*p*_ − 1_ ways to connect *N_p_* − 1 neurons from *P*_1_ to *i* through *n*_1_ synapses (*i* does not self-connect) and Γ*^n_u_^_N_p__* ways to connect *N_p_* neurons from *P_u_* to *i* through *n_u_* synapses (*u* = 2, …, *g*). There are $\Gamma_{N_{E}-gNp}^{N_{es}-\sum_{u=1}^{g}n_{u}}$ ways of connecting the remaining *N_es_* − (*n*_1_ + … + *n_g_*) synapses from the *N_E_* − *gN_p_* NSI-coding neurons to neuron *i*.

Finally, the total number of ways to connect neuron *i* through a dendrite of type [*n*_1_, *n*_2_, …, *n_g_*] is the product of Γ^*n*_1_^_*N*_*p*_ − 1_, Γ^*n*_2_^*_N_p__*, …, Γ*^n_g_^_N_p__* and $\Gamma_{N_{E}-gNp}^{N_{es}-\sum_{u=1}^{g}n_{u}}$. Thus, the probability that a post-synaptic neuron *i* from population *P*_1_ has a dendrite of type [*n*_1_, *n*_2_, …, *n_g_*] is:

(1) $$ProbP_{1}=\left(\left[n_{1,}\ldots ,n_{g}\right]\right)=\frac{\Gamma_{Np-1}^{n_{1}}\;\left(\prod_{u=2}^{g}\Gamma_{Np}^{n_{u}}\right)\;\Gamma_{N_{E}-gNp}^{N_{es}-\sum_{u=1}^{g}n_{u}}}{\Gamma_{N_{g}-1}^{N_{es}}}$$

This result generalizes to the probability that any post-synaptic neuron *i* from any population *P_v_* (1 ≤ *v* ≤ *g*) has a dendrite of type [*n*_1_, *n*_2_, …, *n_g_*]:

(2) $$ProbP_{v}=\left(\left[\text{​}n_{1},\ldots ,n_{g}\text{​}\right]\right)=\frac{\Gamma_{Np-1}^{n_{v}}\text{​}\left(\prod_{u=1,u\neq v}^{g}\Gamma_{Np}^{n_{u}}\text{​}\right)\text{​}\Gamma_{N_{E}-gNp-1}^{N_{es}-\sum_{u=1}^{g}n_{u}}}{\Gamma_{N_{E}-1}^{N_{es}}}\;\;\;$$

and also for NSI-coding neurons:

(3) $$ProbP_{NSI}=\left(\left[n_{1},\ldots ,n_{g}\right]\right)=\frac{\left(\prod_{u=1}^{g}\Gamma_{Np}^{n_{u}}\right)\;\Gamma_{N_{E}-gNp-1}^{N_{es}-\sum_{u=1}^{g}n_{u}}}{\Gamma_{N_{E}-1}^{N_{es}}}$$

This probabilistic law has been verified *via* random number generation (see Figure 1E) and was used for connecting the network for simulations.

### Inter-synaptic learning

During synaptic learning, the local pattern of pre- and post-synaptic activity leads to long-term potentiation (LTP; Hebb, 1949; Bliss and Lomo, 1973; Bliss and Collingridge, 1993) or depression (LTD; e.g., Kirkwood and Bear, 1994) of the synapse. LTP and LTD have been reported with rewarded responses (Soltani and Wang, 2006) resulting from dopamine modulation of synaptic plasticity at prefrontal synapses (Reynolds et al., 2001; Reynolds and Wickens, 2002), while a lack of dopamine signal prevents both LTP (Centonze et al., 1999) and LTD (Calabresi et al., 1992). We therefore consider that learning occurs when the response to a combination of context and stimulus is in accordance with the rule (rewarded combinations). Synapses are updated for each rewarded combination according to the states of the pre- and post-synaptic neurons. Those neurons are active when the context, stimulus or response they code for is involved in the rewarded combination. We first consider the formalism describing Hebbian learning (Brunel et al., 1998) before presenting the new formalism describing IS learning.

#### Hebbian learning

The plastic synapses are assumed to be binary, with two discrete states: a potentiated “Up” state and a depressed “Down” state. The formalism of classical Hebbian learning generates potentiation or depression of synapses as a function of the activity of the two pre- and post-synaptic neurons (e.g., Brunel et al., 1998). For simplicity, we will consider here neurons whose current state *V_i_* ∈ [0; 1] corresponds to its activity in the absence of any external stimulus or context. The state *V_i_* of a neuron *i* is driven by the presence or absence of the item it codes for (context, stimulus or response), described as a binary string ξ_*i*_ ∈ {0; 1}. Learning occurs according to an all-or-none reward signal (depending on the combination) that determines if synapses learn or not for a given trial. When the learning conditions are met, synaptic modification occurs probabilistically (Amit and Fusi, 1994; Brunel et al., 1998; Fusi, 2002; Fusi et al., 2005). In cases of LTP, each synapse in the Down state has an instant probability *q*^+^ to be switched to the Up state. Similarly, in cases of LTD, each synapse in the Up state has an instant probability *q*^−^ of being switched to the Down state. As a result, a synapse *ij* between pre- and post-synaptic neurons *i* and *j* has a probability *a_ij_* to potentiate, a probability *b_ij_* to depress, and probability λ_*ij*_ that no change occurs (Brunel et al., 1998):

(4) $$a_{ij}=q^{+}\xi_{i}\xi_{j}$$

(5) $$b_{ij}=q^{-}\left[\xi_{i}\left(1-\xi_{j}\right)+\xi_{j}\left(1-\xi_{i}\right)\right]$$

(6) $$\lambda_{ij}=1-a_{ij}-b_{ij}$$

#### Inter-synaptic amplification of potentiation

In our model, we consider that the spatial organization of synapses in the dendritic tree impacts the integration of synaptic inputs within the dendrites. Dendritic compartmentalization influences the pairing of excitatory post-potentials (EPSP) generated in dendrites and action potentials (see Spruston, 2008). Studies have reported that the induction of LTP requires a minimal amount of synapse activation (Govindarajan et al., 2011), due to the activation of at least some biochemical pathways that are spread over a short distance (Yasuda et al., 2006; Harvey et al., 2008) and/or the electrical supralinear summation of synaptic inputs within subdendritic domains (Poirazi et al., 2003a,b; Gasparini et al., 2004). Synapses at which LTP has been induced can then benefit from further LTP when other synapses are potentiated through the use of plasticity-related protein products (Frey and Morris, 1997). The clustered plasticity hypothesis (Govindarajan et al., 2006) predicts that, based on local activity-induced protein synthesis (Steward and Schuman, 2001; Martin and Kosik, 2002), potentiation is amplified for synapses that are close in a dendritic branch (see Harvey and Svoboda, 2007). This is in line with the observation of LTP within the same dendritic branches rather than across branches (Govindarajan et al., 2011). Moreover, these authors also report that, under conditions in which spines are located within the same dendrite, the number of spines that are potentiated increases with the number of spines that are stimulated (Govindarajan et al., 2011 Figure 6).

In our model, we consider the number of active synapses within the dendrite to be of critical importance in the amplification of their potentiation, i.e., the potentiation of a given synapse between a post-synaptic and a pre-synaptic neuron is amplified when other pre-synaptic neurons having synapses within the same dendrite are also activated (active synapses). The resulting inter-synaptic learning rule has been formalized according to a mathematically tractable description of the average potentiation values of the synapses in the different types of dendrites after a learning protocol of any type of combination rule.

We will next consider a dendrite *D* of type [*n*_1_, *n*_2_, …, *n_g_*]. From Equation 4, the probability *a_ij_*(*D*) of potentiating an active synapse connecting two active neurons *i* and *j* within a dendrite *D* is equal to its instant probability *q*^+^ of being switched to the Up state. Here, we consider that the probability *a_ij_*(*D*) will increase according to the number of other active synapses in *D*. The simplest way to take this in account is to multiply *q*^+^ by *n*:

(7) $$a_{ij}\left(D\right)=q^{+}\xi_{i}\xi_{j}n$$

We note here that *a_ij_*(*D*) ≤ 1 corresponds to cases of small values of *q*^+^ (slow learning; Brunel et al., 1998) and of a small number *n* of active synapses in a given dendrite *D*. Here we take *q*^+^ = 0.01 and the maximum number of active synapses per dendrite *N_s_* = 16.

If the population of neurons *P_u_* is inactive/active, every neuron that belongs to it is, respectively, inactive/active. By extension, we will denote ξ_*Pu*_ ∈ {0; 1} the state of every neuron in that population.

Let us now consider a dendrite of type [*n*_1_, *n*_2_, …, *n_g_*], of a post-synaptic neuron *i* and consider that a pre-synaptic neuron *j* belongs to population *P_j_* such that the synapse *ij* is one of the *n_j_* synapses from population *j* within the dendrite. The number of active synapses from population *P_u_* is ξ*_Pu_n_u_*, and thus the number of active synapses *n* within the dendrite can be decomposed as:

(8) $$n=\xi_{Pj}n_{j}+\sum_{u=1,\;u\neq j}^{g}\xi_{Pu}n_{u}$$

As ξ_*Pj*_ = ξ_*j*_ Equation (7) can be now written as:

(9) $$a_{ij}\left(D\right)=q^{\;+\;}\xi_{i}\xi_{j}+q^{\;+\;}\xi_{i}\xi_{J}\text{​}\left(n_{j}-1\right)+q^{\;+\;}\xi_{i}\xi_{j}\left(\sum_{u=1,\;u\neq j}^{g}\xi_{Pu}n_{u}\right)\text{​​​​}$$

with *q*^+^ ξ_*i*_ ξ_*j*_ representing the synaptic potentiation due to classic Hebbian learning (Equation 4), *q*^+^ ξ_*i*_ξ_*J*_(*n_j_* − 1) representing the inter-synaptic amplification of the potentiation due to co-active synapses connecting neurons from the same population *P_j_*, and $q^{\;+\;}\xi_{i}\xi_{j}\left(\sum_{u=1,\;u\neq j}^{g}\xi_{Pu}n_{u}\right)$ representing the inter-synaptic amplification of potentiation due to co-active synapses connecting neurons from different populations *P_u_*. The amplification of potentiation of a given synapse depends on the activity of the post-synaptic neuron, of the pre-synaptic neuron, and of other active neurons having a synaptic contact within the same dendrite *D*. Synaptic potentiation is calculated locally within a dendrite, but obeys a non-local rule that takes into account the activity of neurons other than the pre- and post-synaptic neuron.

#### Inter-synaptic amplification of depression

The probability *b_ij_* of depressing a synapse connecting two active neurons *i* and *j* within a dendrite *D* is equal to its instant probability *q*^−^ of being switched to the Down state if either *i* is active and *j* is inactive or *i* is inactive and *j* is active (Equation 5). Here, we consider that the probability *b_ij_*(*D*) will depend on the number of other active synapses in *D*: *b_ij_* increases according to the number of others active synapses in *D* if *i* is active and *j* is inactive, or if *i* is inactive and *j* is active. As in the case of amplification of potentiation, the simplest way to take that increase into account is by multiplication. Considering a dendrite *D* of type [*n*_1_, *n*_2_, …, *n_g_*] we thus have:

(10) $$b_{ij}\left(D\right)=q^{-\;}\xi_{i}\left(1-\xi_{j}\right)\left(1+\sum_{u=1,u\neq j}^{g}\xi_{Pu}n_{u}\right)+q^{-\;}\xi_{j}\left(1-\xi_{i}\right)n_{j}$$

We also note here that *b_ij_*(*D*) < 1 corresponds to cases of small values of *q*^−^ (slow learning; Brunel et al., 1998) and of a small number *n* of active synapses in a given dendrite *D*. Here we take *q*^−^ = *q*^+^ = 0.01 (the maximum number of synapses per dendrite *N_s_* = 16).

Equation 10 can be rewritten:

(11) $$\begin{array}{l} b_{ij}\left(D\right)=q^{-\;}\xi_{i}\left(1-\xi_{j}\right)+q^{-\;}\left(1-\xi_{i}\right)\xi_{j}+q^{-\;}\text{​}\xi_{j}\text{​}\left(1-\xi_{i}\right)\text{​}\left(n_{j}-1\right)\\ \;+\;q^{-\;}\xi_{i}\left(1-\xi_{j}\right)\sum_{u=1,u\neq j}^{g}\xi_{Pu}n_{u}\text{​​​​​​} \end{array}$$

with *q*^−^ ξ_*i*_ (1 − ξ_*j*_) + *q*^−^(1 − ξ_*i*_) ξ_*j*_ representing the synaptic depression due to classic Hebbian learning (Equation 5), *q*^−^ ξ_*j*_ (1 − ξ_*i*_)(*n_j_* − 1) representing the inter-synaptic amplification of depression due to co-active synapses connecting neurons from the same population *P_j_*, and *q*^−^ ξ_*i*_ (1 − ξ_*j*_)$\sum_{u=1,\;u\neq j}^{g}\xi_{Pu}n_{u}$ representing the inter-synaptic amplification of depression due to co-active synapses connecting neurons from different populations *P_u_*. The amplification of depression of a given synapse depends on the activity of the post-synaptic neuron, of the pre-synaptic neuron, and of other active neurons having a synaptic contact within the same dendrite *D*. Like synaptic potentiation, synaptic depression is calculated locally within a dendrite, but obeys a non-local rule that takes into account the activity of other neurons than the pre- and post-synaptic neuron.

#### No change

From Equation 6, the probability that a synapse does not change can be written as follows for a given dendrite:

(12) $$\lambda_{ij}\left(D\right)=1-a_{ij}\left(D\right)-b_{ij}\left(D\right)$$

#### Overall probability of potentiation

According to Brunel et al. (1998), *a_ij_* and *b_ij_* allow the calculation of the mean values of potentiation *J_ij_* as the mean probability of potentiating the synapse *ij* without further changes along the learning protocol, under the assumption that learning is slow (i.e., *q*+ and *q*− are low; see Brunel et al., 1998). *J_ij_* is the mean probability reaching the value *a_ij_*, for each presentation of the stimuli ξ_*i*_ and ξ_*j*_ at each discrete time *t* (from *1* to the last learning time *T*), and that the value *a_ij_* does not change (λ_*ij*_) at each time *t* afterward (from *s* = *t* + 1 to *T*):

(13) $$j_{ij}\left(T\right)=\sum_{t=1}^{T}a_{ij}(t)\prod_{s=t+1}^{T}\lambda_{ij}(s)$$

If we consider a dendrite *D* of type [*n*_1_, *n*_2_, …, *n_g_*], we can calculate the mean values of potentiation *J_ij_* as the mean probability of potentiating the synapse *ij* without further changes for each type of dendrite (*a_ij_* from Equation 9 and λ_*ij*_ from Equation 12):

(14) $$j_{ij}\left(D,T\right)=\sum_{t=1}^{T}a_{ij}\left(D,t\right)\prod_{s=t+1}^{T}\lambda_{ij}\left(D,s\right)$$

Given that each of the *a^t^_ij_*Π*_s_*λ*^s^_ij_* is a product of terms corresponding to different times, they can be averaged independently since presentations at different time steps are uncorrelated. According to Brunel et al. (1998), we obtain, for each type of dendrite, the average probability *J_ij_* that a synapse is potentiated after the presentation of all combinations of items in all possible orders (case of infinite and slow learning):

(15) $$J_{ij}\left(D\right)=\langle J_{ij}\left(D\right)\rangle =\langle a_{ij}\left(D\right)\rangle \sum_{s=0}^{\infty }\lambda_{ij}^{\left(D,s\right)}=\frac{\langle a_{ij}\left(D\right)\rangle }{\langle a_{ij}\left(D\right)+b_{ij}\left(D\right)\rangle }$$

*J_ij_* is the probability that a synapse connecting a post-synaptic neuron *i* and a pre-synaptic neuron *j* is potentiated. It corresponds to the probability *a* in the notation of Brunel et al. (1998), and allows us to calculate the exact value of synaptic efficacy *J_a_* (see Mongillo et al., 2003):

- Synapses between neurons coding for different and associated items have an intermediate efficacy *J_a_* whose value depends on the probability *a*, which depends in turn on the type of dendrite considered and to the IS learning algorithm:
  (16) $$J_{a}=J_{0}+a(J_{1}-J_{0})$$
- Synapses between neurons coding for a same item have a maximum efficacy *J_1_* that corresponds to the maximum probability *a* = 1:
  (17) $$J_{1}=k.J_{EE}$$
  with *J_EE_* the value of efficacy before learning, and *k* = 2.09 so that neurons coding for the same item can exhibit persistent activity after removal of the stimulus (see Amit and Brunel, 1997).
- Synapses between neurons coding for different and non-associated items, or between coding and NSI neurons, have minimum efficacy *J_0_*:
  (18) $$J_{0}=(J_{EE}-fJ_{1})/(1-f)$$
  with *f* being the coding level and *J_EE_* being the average efficacy of excitatory to excitatory synapses.

The IS learning algorithm allows the calculation of the synaptic efficacy of all the synapses as a function of their probability of being potentiated according to each type of dendrite. The combinations of items are learned solely through the modification of synaptic efficacy, which depends on the states of the neurons coding for the items during the learning of a combination, i.e., on the context, stimulus, and response involved in that combination.

### Dendritic and neuronal dynamics

#### Dendritic dynamics

Dendrites integrate synaptic currents induced by AMPA (α-amino-3-hydroxy-5-methyl-4-isoxazolepropionic acid) and NMDA (N-methyl-D-aspartate) receptors to glutamate, and currents induced by GABA (γ-aminobutiric acid) receptors to GABA. This allows us to calculate voltage-independent AMPA and GABA synaptic currents and voltage-dependent NMDA currents obeying their own dynamics. The total dendritic current *I_D_* is a composite of different currents *I^R^*, corresponding to different types of post-synaptic receptors *R* to GABA, AMPA and NMDA.

Upon the emission of a pre-synaptic spike at *t_k_*, an epsp/ipsp is generated within the dendrite *D* after a delay δ_*s*_. The current has an instantaneous jump proportional to the efficacy *Js* (mS) of the synapse *s*. It is followed by an exponential decay with a time constant τ^*R*^. Different τ^*R*^ values correspond to the different receptors involved (Hestrin et al., 1990; Spruston et al., 1995; Salin and Prince, 1996; Xiang et al., 1998): GABARs exhibit fast activation and decay (τ^*GABA*^ = 5 ms), AMPARs exhibit fast activation and decay (τ^*AMPA*^ = 2 ms), and NMDARs exhibit slow activation and decay (τ^*NMDA*^ = 100 ms).

For GABA receptors, voltage-independent inhibitory post-synaptic currents *I*^*GABA*^ obey the equation:

(19) $$\tau^{GABA}\frac{dI_{D}^{GABA}\left(t\right)}{dt}=-I_{D}^{GABA}\left(t\right)+\tau_{m}\sum_{s}J_{s}\sum_{k}\delta \left(t-t_{k}-\delta_{s}\right)$$

For AMPA receptors, voltage-independent excitatory post-synaptic currents *I^AMPA^* obey the equation:

(20) $$\begin{array}{l} \tau^{AMPA}\frac{dI_{D}^{AMPA}\left(t\right)}{dt}=-I_{D}^{AMPA}\left(t\right)+\tau_{m}\sum_{s}\left(1-x\right)J_{s}\\ \;\sum_{k}\delta \left(t-t_{k}-\delta_{s}\right) \end{array}$$

where (*1* − *x*) is the fraction of excitatory currents induced by AMPA receptors, and *x* is the fraction of excitatory currents induced by NMDA receptors.

NMDA receptors are voltage-dependent. Excitatory post-synaptic currents *I^NMDA^_d_* are calculated as a function of the dendrite potential *V_D_* according to the equation:

(21) $$\begin{array}{l} \tau^{NMDA}\frac{dI_{D}^{NMDA}\left(t\right)}{dt}=-I_{D}^{NMDA}\left(t\right)+\frac{\tau_{m}}{1+\left(\frac{1}{3.57}\right)e^{-0.062V_{D}\left(t\right)}}\\ \;\sum_{s}xJ\sum_{k}\delta \left(t-t_{k}-\delta_{s}\right) \end{array}$$

The dendritic currents generated by each receptor *R* in each dendrite *D* are due to recurrent excitatory and inhibitory activities and to external noise and input stimuli. Within each dendrite *D*, currents evolve with their own dynamics, with the GABA_*A*_ and AMPA currents being linearly integrated.

Non-linear dendritic integration relies on NMDA currents that are non-linearly integrated within each dendrite before arriving at the cell body. NMDA currents vary according to the multiplying factor *S_I_* (*I^NMDA,rec^_d_*) that varies non-linearly with *I^NMDA,rec^_d_* according to a sigmoïd between the values 1 and 1 + *G*:

(22) $$S_{I}\left(I_{D}^{NMDA}\right)=1+\frac{G}{1+e^{\frac{\gamma -\left|I_{D}^{NMDA}\right|}{s}}}$$

with $\gamma =\frac{I_{max}+I_{min}}{2},s=\frac{\left|I_{max}-I_{min}\right|}{20}.G=12.5$ is the gain and *I_min_* and *I_max_* are the minimum and maximum values of dendritic current, respectively (see Table 1).

**Table 1:** **Description and parameters for the model**.

**MODEL SUMMARY**		
Populations	One of inhibitory neurons	
	Six of excitatory neurons (selective to a context, stimulus or response)	
	One of NSI-coding excitatory neurons (not selective to a context, stimulus or response). A fraction can respond to combinations (mixed-coding neurons, depending on simulations)	
Connectivity	Random	
Neuron model	Leaky integrate and fire, fixed voltage threshold, fixed absolute refractory period	
Dendrite model	Linear integration of GABA and AMPA currents. Voltage dependent linear or non-linear integration of NMDA currents (depending on simulations)	
Synapse model	δ-current inputs (discontinuous voltage jumps)	
Plasticity	Hebbian or Inter-Synaptic Learning (depending on simulations)	
Input	Independent fixed-rate Poisson spike trains to neurons coding for the context, stimulus or response	
Measurements	Averaged spike frequencies of the neurons of each population	
**CONNECTIVITY**		
*N_E_*	Number of excitatory neurons	4000
*N_I_*	Number of inhibitory neurons	1000
*C*	Connectivity	0.2
*C_E_*	Number of recurrent excitatory connections per neuron	800
*C_ext_*	Number of external excitatory connections per neuron	2200
*C_I_*	Number of recurrent inhibitory connections per neuron	200
*g*	Number of populations	6
*fN_E_*	Number of neurons per population	400
*f*	Coding level	*N_Ep_/N_E_* = 0.1
*N_s_*	Number of excitatory synapses per dendrite	16
*N_d_*	Number of dendrites per neuron	50
**DYNAMICS**		
δ_*E*_	Latency (transmission delay), excitatory neurons	15–30 ms
δ_*I*_	Latency (transmission delay), inhibitory neurons	0.5 ms
*τ_AMPA_*	Synaptic decay type, AMPA-R	2 ms
*τ_NMDA_*	Synaptic decay type, NMDA-R	100 ms
*τ_GABA_*	Synaptic decay type, GABA-R	5 ms
*x*	Fraction of NMDA currents	0.3
*τ_mE_*	Membrane time constant, excitatory neurons	20 ms
*τ_mI_*	Membrane time constant, inhibitory neurons	10 ms
θ	Firing threshold, both types	20 mV
*V_τ__E_*	Reset membrane potential, excitatory neurons	10 mV
*V_τ__I_*	Reset membrane potential, inhibitory neurons	15 mV
*τ_RP_*	Refractory period, both types	2 ms
λ	Contrast of external input	0.08
*I_min_*	Minimum value of dendritic current	0
*I_max_*	Maximum value of dendritic current	0.115
*ν_ext_*	External Poisson noise	15 Hz
**SYNAPSES AND LEARNING**		
*q+*	Intrinsic probability of potentiation	0.01
*q−*	Intrinsic probability of depression	0.01
*J_EE_*	Average E→E efficacy	0.047 mV
*J_IE_*	E→I efficacy	0.09 mV
*J_EI_*	I→E efficacy	−0.27 mV
*J_II_*	I→I efficacy	−0.5 mV
*J_Eext_*	External E→E efficacy	0.052 mV
*J_Iext_*	External E→I efficacy	0.1 mV
*J_1_*	Potentiated E→E efficacy between neurons coding for a same item	0.098
*J_a_*	Potentiated E→E efficacy between associated items	*J_0_ + a(J_1_ − J_0_)*
*J_0_*	Depressed E→E efficacy between non-associated items	*(J_EE_ − fJ_1_)/(1 − f)*

NMDA currents are then multiplied by *S_I_*(*I^NMDA,rec^_d_*) and therefore vary non-linearly. In some simulations testing the effects of linear dendritic integration, *S_I_*(*I^NMDA,rec^_d_*) will be set to 1 (see Results—Selectivity and Responsiveness of the Different Types of Dendrites).

All receptor-dependent currents are then summed within the dendrite, to give the dendritic current *I_D_*:

(23) $$I_{D}=S_{I}\left(I_{D}^{NMDA}\right)I_{D}^{NMDA}+I_{D}^{AMPA}+I_{D}^{GABA}+\left(1+\lambda \right)I_{D}^{AMPA,ext}$$

where *I^AMPA,ext^_D_* is the external current induced by noise which we assume to be induced by AMPA receptors only. λ is the contrast of the external afferent input over external noise, and is equal to 0 when no input is presented to the network and equals 0.08 for a given neuron population when the neuron receives selective afferents, when the specific item is presented to the network with a rate (1+λ)ν_*ext*_ (Mongillo et al., 2003).

The dendritic potential *V_D_* is calculated in each dendrite *D* as a function of the synaptic current *I_D_* in the dendrite (in units of *V_D_*), generated by spikes arriving from pre-synaptic neurons.

(24) $$\tau_{m}\frac{dV_{D}\left(t\right)}{dt}=-V_{D}\left(t\right)+I_{D}\left(t\right)$$

where *τ_m_* is the membrane time constant of excitatory cells (*τ_E_* = 20 ms) and inhibitory cells (*τ_I_* = 10 ms).

#### Neuronal dynamics

Each neuron *i* of the network is a leaky integrate-and-fire neuron (Tuckwell, 1988), whose state is described by its total depolarization *V* (mV) and is calculated as follows:

(25) $$\tau_{m}\frac{dV\left(t\right)}{dt}=-V\left(t\right)+\sum_{D}I_{D}\left(t\right)$$

For simplicity, we consider that the integration in a given dendrite is independent of that in other dendrites, and that all dendrites have the same weight when summed in the cell body. When *V* reaches a threshold *V^θ^*, the neuron emits a spike and *V^T^* is reset to *V_τ_*, following a refractory period *τ_RP_*.

## Results

The main objective of the present formalism of IS learning is to investigate how important a role the interactions between nearby synapses within dendrites play at the stages of learning and of processing of XOR-like combinations at the network level. The results section is organized as follows: Section Probability of the Different Types of Dendrites presents a description of the types of dendrites generated by random connectivity; Section Inter-Synaptic Learning in the Different Types of Dendrites presents the IS learning of the values of synaptic efficacy depending on the number of co-active synapses that are present in different types of dendrites, as well as a comparison of IS learning and classical Hebbian learning; Section Selectivity and Responsiveness of the Different Types of Dendrites presents the effects of IS learning on the dendritic response to combinations of contexts and stimuli, and the role of non-linear dendritic integration in the amplification of the learned combinations; and Section Neuron and Network Processing of XOR-like Combinations presents the behavior of the network processing a XOR-like combination after IS learning and a comparison to Hebbian learning and the synergistic effects of IS learning and mixed-coding neurons.

### Probability of the different types of dendrites

**Figure 3:**
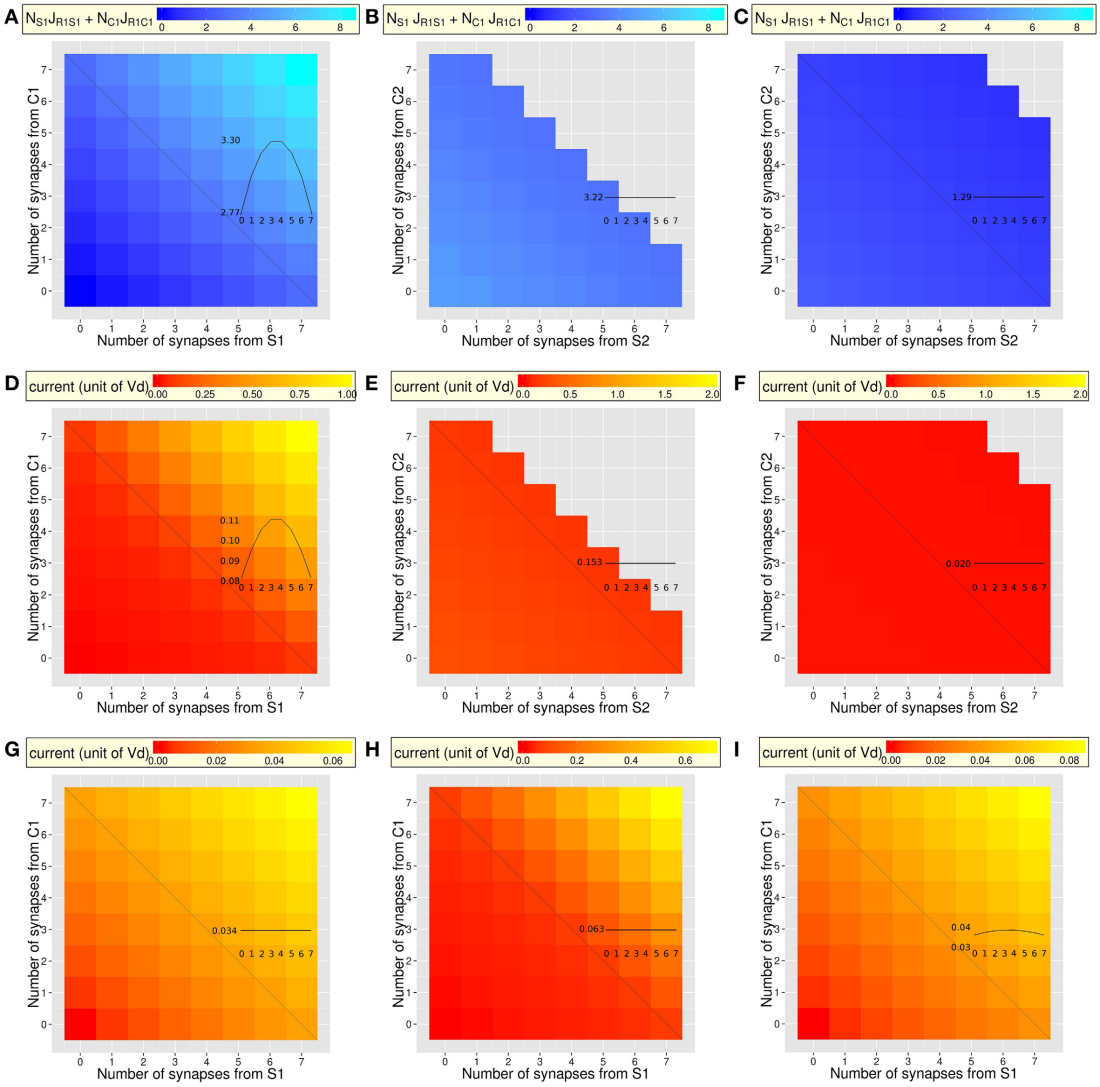
**Selectivity and responsiveness of the different types of dendrites to the combined activity of the context and stimulus (same dendrites as in Figure 2)**. Insets display the total synaptic input or dendritic current along the anti-diagonal of each figure (see Figure 2). **(A–C)** Total pre-synaptic input, as the sum of two efficacies weighted by their number (*n_S_J*_*R*1−*S*_ + *n_C_J*_*R*1−*C*_). **(A)** Amplification of the total synaptic input increases with amplification of potentiation and with the number of synapses (mean = 3.40; min = 0; max = 8.55). Along the anti-diagonal, synaptic input varies with the ratio of numbers of synapses from C2 and S2, even though the number of synapses is constant. **(B,C)**. Overall synaptic input is stronger in [C1 = 4, S1 = 4, C2, S2, R1, R2] dendrites (**B;** mean = 3.58; min = 3.05; max = 4.69) than in [C1 = 2, S1 = 2, C2, S2, R1, R2] dendrites (**C;** mean = 1.34; min = 0.84; max = 2.16). In both cases, synaptic depression decreases efficacy with the increasing number of synapses from C2 and S2 that generate depression of the C1R1 and S1R1 synapses when learning of a C2S2R1 combination. **(D–I)** Total dendritic current *I_D_* generated by a spike train of 20 Hz on synapses from neurons coding for the context and stimulus S1 and S2 (averaged over a 100 ms stimulation). **(D–F)** Non-linear integration following IS learning (same dendritic types as A-C). **(D)** Currents greatly increase with the number of potentiated synapses from C1 and S1 (mean = 0.238; min = 0; max = 1.03). The non-linear responses of dendrites magnify the effect of the efficacy (compare with Figure 2D). Dendritic currents also vary along the anti-diagonal for a constant number of synapses. **(E,F)** Two types of dendrites having [4C1, 4S1] or [2C1, 2S1] synapses (same as Figures 2E,F) further show the increased dendritic current as a function of the number of synapses from C2 and S2. The overall dendritic current is stronger in [C1 = 4, S1 = 4, C2, S2, R1, R2] dendrites (**E;** mean = 0.18; min = 0.14; max = 0.28) than in [C1 = 2, S1 = 2, C2, S2, R1, R2] dendrites where amplification of potentiation is too weak to generate different currents in the different dendrites (**F;** mean = 0.02; min = 0.02; max = 0.02). When dendrites have too few synapses from C1 and S1, they lose responsiveness to the C1S1 combination **(C)**. **(G)** Same as **(D)** in case of linear dendritic integration following classic Hebbian learning: currents vary in very small amount due to linear dendritic integration (mean = 0.03; min = 0, max = 0.07, compare with **(D)**, with a different scale). Along the anti-diagonal, for a constant number of linearly integrated inputs, currents do not vary with the ratio of numbers of synapses from C2 and S2 due to the absence of IS learning. **(H)** Same as **(D)** in case of non-linear dendritic integration and classical Hebbian learning: currents vary in larger amount than in G but smaller than in **(D)** (mean = 0.15; min = 0; max = 0.72) due to non-linear integration. As in **(G)**, dendritic currents do not vary along the anti-diagonal due to the absence of IS learning. **(I)** Same as **(D)** in case of linear dendritic integration and IS learning: Currents vary in small amount across dendrites (mean = 0.04; min = 0; max = 0.09: compare with **D**). The anti-diagonal exhibits the pure effect IS learning, with dendritic currents that vary even though dendritic integration is linear (see Figure 2D).

Given that all dendrites have the same, small number *N_s_* of synapses compared to the total number of potential pre-synaptic contacts, random connectivity generates dendrites that have different numbers of synapses with pre-synaptic neurons coding for the different contexts, stimuli, or responses (from zero to the maximum number of synapses *N_s_* in the dendrite). Some of the dendrites will have more synapses for a given context, stimulus and response, and will thus be better able to respond to their particular combination. At the level of neurons and of the network, it is therefore necessary to know the probability of each type of dendrite. We first analyze the random distribution of synapses in the different types of dendrites involved (Figures 2A–C; Methods—Synaptic Connectivity; Equation 2 and 3). The probability of occurrence of each type of dendrites is highly variable (Figure 2A). It is heterogeneous even for a fixed number of synapses from C1 and S1 (along the anti-diagonal; that will show the effects of IS learning for a fixed number of synapses; Figures 2, 3). The probability is also heterogeneous depending on the number of synapses from C2 and S2 within two relevant types of dendrites: the rare case of four synapses from C1 and four from S1 ([C1 = 4, S1 = 4, C2, S2, R1, R2], Figure 2B) and the frequent case of two synapses from C1 and two from S1 ([C1 = 2, S1 = 2, C2, S2, R1, R2], Figure 2C). The different probabilities of the different types of dendrites creates the possibility that only a fraction of the dendrites have more synapses from neurons coding for combinations that will be learned (e.g., for a dendrite on a neuron coding for R1: dendrites having synapses from C1S1 and/or C2S2). The result is that random connectivity at the level of dendrites having few synapses makes different dendrites with different numbers of synapses from the different contexts, stimuli, and response. These dendrites are therefore more or less susceptible to learn some specific combinations through IS learning.

### Inter-synaptic learning in the different types of dendrites

The next step is to determine, for a given type of synapse (e.g., between C1 and R1 neurons), to what extent IS learning generates different efficacy values in different types of dendrites with different numbers of synapses from pre-synaptic neurons coding for items combined together (e.g., C1S1R1). Finally, we compare the effect of IS learning (Equation 15) to the effect of classical Hebbian learning (see Brunel et al., 1998) on the synaptic efficacy values in the different types of dendrites.

Considering a specific set of synapses that connect neurons coding for a given pair of context and stimulus (C1 & S1) to a response (R1), the results show that the inter-synaptic learning algorithm generates, for a given pair of synapses (e.g., S1-R1 and C1-R1), efficacy values that depend greatly on the number of this type of synapses in the dendrite (Figure 2D; Methods—Synaptic Connectivity and Inter-synaptic Learning). The different efficacy values generated by IS learning in the different types of dendrites correspond to a synaptic efficacy mosaic (Iannella et al., 2010).

An important point is visible on the types of dendrites along the diagonal of the matrix (Figure 2D), where the efficacy of two synapses (from C1 and from S1) increases with the number of synapses from C1 and from S1 within the dendrite. Regarding the anti-diagonal of Figure 2D, the curve—corresponding to the sum of the efficacy of two synapses C1-R1 and S1-R1—exhibits a concave shape (inset of Figure 2D). This is due to the fact that, along the anti-diagonal, the amplification of potentiation of a synapse C1-R1 alone increases with the increasing number of synapses from C1 (inset of Figure 2G), while the amplification of potentiation of a synapse S1-R1 alone increases in the opposite direction with the increasing number of synapses from S1. The combination of the two phenomena leads to the concave curve of Figure 2D (inset). An interesting feature is that, when multiplied by the number of synapses of each type in the dendrite, the shape of the curve becomes convex (Figure 3A) and predicts different amounts of dendritic currents (Figure 3D).

Figure 2D shows that synaptic amplification of potentiation is larger in the rare dendrites having four synapses from C1 and four from S1 ([C1 = 4, S1 = 4, C2, S2, R1, R2], see Figure 2E) than in the frequent dendrites having two synapses from C1 and two from S1 ([C1 = 2, S1 = 2, C2, S2, R1, R2], see Figure 2F). IS learning of combinations therefore involves rare dendrites that are more selective to those combinations. To examine the effects of amplification of synaptic depression, efficacies are further examined in these dendrites as a function of the number of synapses from C2 and S2 that are learned in combination with R1 and depress synapses from C1 and S1. For these two types of dendrites, Figures 2E,F show that an increasing number of synapses from either C2 or S2 decreases the efficacy of synapses from C1 and S1. This shows the effects of the amplification of synaptic depression due to the other learned combination C2S2R1, which depresses synapses from C1 and S1 proportionally to the number of synapses from C2 and S2. Note the flat curves along the anti-diagonals (insets of Figures 2E,F) that show that efficacy of C1R1 and S1R1 synapses does not depend on the number of synapses from neurons coding for items not combined with R1 and C1 or S1 and R1 (here C2 and S2). Note also the highest efficacy when more synapses from C1 and S1 are present in the dendrite (Figure 2E compared to Figure 2F; visible on the y-value of the insets).

Figure 2H shows the effects of synaptic depression on synapses R1-C2 from a context (C2) that is not learned in combination with the stimulus and response (S1R1). This is to compare to synapses R1C1 from a context (C1) that is learned in combination with the same stimulus and response (S1R1; Figure 2G). When comparing to classical Hebbian learning (Figure 2I and inset), the Hebb rule generates strong potentiation of synapses that connect neurons coding for the contexts, stimuli and responses, regardless of the number of co-active synapses within the dendrite. Hebbian learning does not generate different efficacy values for a given type of synapse although the algorithm is applied at the level of dendrites and not of point neurons. This is in clear contrast with the amplification of potentiation and depression generated by IS learning (Figure 2D) and reveals the role not only of the synapses localization but of the IS algorithm itself. Synapses are grouped within dendrites, while being in different dendrites than other groups of synapses. Given that groups of synapses in dendrites are small, different dendrites have different numbers of a given type of synapse (e.g., between S1 and R1). As a consequence, IS learning generates efficacy values of a given type of synapse that are different from dendrite to dendrite. This would not be possible by applying a classical Hebbian algorithm, because it does not take into account of the number of active synapses and would generate the same efficacy value for all synapses of a given type whatever the dendrite considered. Further, IS learning applied in the absence of different dendrites would not work either (i.e., by considering a point neuron or a single dendrite grouping all the synapses to the neuron). In the absence of different dendrites, IS learning would take into account of the total number of active synapses to the neuron and not those that are coactive within the same dendrite. This total number being constant if synapses are not grouped in different dendrites, IS learning would generate the same synaptic efficacy for all synapses of a same type. Considering a neurons coding for R1, potentiation of all synapses from neurons coding for C1, C2, S1 or S2 would be amplified in the same amount, because synapses from C1 and S1, or from C2 and S2 would be equally co-active. As a result of either Hebbian learning with different dendrites or of IS learning without dendrites, equal values of synaptic efficacy would not allow the network to discriminate C1S1, C1S2, C2S1, or C2S2 combinations.

To summarize, the amplification of potentiation and depression is determined by the inter-synaptic learning algorithm, which is applied to different numbers of co-active synapses in the different types of dendrites (diagonal of Figure 2D). Further, for dendrites having a constant number of synapses from a context and a stimulus, IS learning generates different efficacy values depending on the ratio of the number of synapses from the context and from the stimulus (inset of Figure 2D). Therefore, though synaptic connectivity is random, different types of dendrites of a neuron coding for a response learn certain combinations rather than others, with an optimum when the numbers of synapses from the context and from the stimulus are equal (i.e., along the diagonal). Here, optimal learning of particular combinations arises at the level of individual dendrites that code for associations between a given stimulus and response in a given context (Figure 2G), but not in another context (Figure 2H).

### Selectivity and responsiveness of the different types of dendrites

The fact that IS learning generates different efficacy values in different dendrites does not necessarily mean that this will significantly change the dendritic responses necessary to optimize the behavior of the network. We investigate here to what extent the total synaptic input to dendrites—defined here by the synaptic efficacy of active synapses—determines the magnitude of dendritic currents (Figures 3A–C). We also compare the effects of non-linear to linear integration of combinations of synaptic inputs on dendritic currents (Figures 3D–I).

The selectivity of the different types of dendrites to particular combinations of synaptic inputs is described by the total synaptic input, as measured by the sum of synaptic efficacies multiplied by their number (Figures 3A–C). Regarding a neuron coding for R1, the total input in the dendrite increases naturally with the number of synapses from C1 and S1 within the dendrite (Figure 3A). As a consequence, non-linear dendritic integration generates currents that increase non-linearly with the amount of synaptic input, being larger in dendrites having four synapses from C1 and four from S1 than in dendrites having two synapses from C1 and two from S1 (Figure 3D). It is important to note here that the total input, as well as the dendritic current, varies also along the anti-diagonal with the ratio of the numbers of synapses from C1 and from S1 while their total number remains constant (convex curve in the inset of Figures 3A,D). This variation along the anti-diagonal arises from the product of the efficacy of each type of synapse by its number within the dendrite. This reveals that, after IS learning, dendrites (e.g., of a neuron coding for R1) are more or less selective to some combinations as a function of the number of synapses from C1 and from S1 (diagonal), but also vary in selectivity for a fixed number of synapses, as a function of the ratio of their numbers (Inset of Figure 3A).

Regarding type of dendrites receiving the largest total input (Figure 3B), responsiveness decreases when the number of synapses from either C2 or S2 increases (diagonal of Figure 3E). This is due to lower synaptic efficacy (due to synaptic depression, Figures 2E). Regarding dendrites receiving fewer synaptic inputs (Figure 3C), responsiveness stays very weak whatever the number of synapses from C2 and from S2 (Figure 3F), due to low efficacy from C1 and from S1 (Figure 2F).

These results show that the non-linear response of dendrites is selective to some combinations of synaptic inputs after IS learning. The synergistic effects of non-linear dendritic integration and IS learning are next investigated by cross-manipulating the type of dendritic integration used (linear vs. non-linear) and the choice of either Hebbian vs. IS learning. Non-linear integration is calculated using Equation 23 by taking *S_I_*(*I^NMDA^_D_*) of Equation 22. Linear integration is calculated by setting *S_I_(I^NMDA^_D_) = 1* and taking *I^NMDA^_D_* according to Equation 21. Figure 3D shows the combined effects of non-linear dendritic integration and IS learning on the amplification of dendritic currents with the increasing number of combined synaptic inputs. To the opposite, Figure 3G shows that in case of linear dendritic integration and Hebbian learning, the responsiveness of dendrites is very weak and exhibit negligible variations due to the increasing number of synapses along the diagonal (note the different scales in Figures 3D,G). Along the anti-diagonal, dendritic currents do not vary for a fixed number of synapses from C2 and S2 (inset of Figure 3G). This is due to the Hebbian learning that cannot change synaptic efficacy with the ratio of numbers of synapses from C2 and S2. Figure 3H shows that, when non-linear dendritic integration is allowed but not IS learning, currents vary in larger amount with the number of synapses along the diagonal, due to the sole effect of non-linear integration. However, along the anti-diagonal, synaptic efficacy does not change with this number due to the absence of IS learning. Finally, Figure 3I shows that, when dendritic integration is only linear and IS learning is allowed, currents increase in small amounts with increasing number of synapses along the diagonal. The pure contribution of IS learning is visible along the anti-diagonal, where dendritic currents exhibit a convex curve. However, the convexity is amplified by non-linear dendritic integration (Figure 3D). Non-linear integration is necessary for a neuron to discriminate between two learned combinations, even in case of IS learning. Let us consider a dendrite with two groups of synapses from a context and a stimulus (C1 and S1) and another dendrite with two other groups (C2 and S2). According to the rule, both groups are combined with the response (here R1 coded by the post-synaptic neuron) and synapses all have the same efficacy due to amplification of potentiation. A consequence of this is that, in the case of linear dendritic integration, every combination of inputs (e.g., C1S1, C1S2, C2S1, C2S2) would be linearly integrated before arriving at the cell body, regardless of their dendrite of origin. In the case of non-linear integration, only combinations within the same dendrite (e.g., C1S1 and C2S2) would be non-linearly integrated and lead to amplification of the current. Other combinations arising from different dendrites would be simply summed before arriving at the cell body. It is thus the combination of IS learning and non-linear dendritic integration that allow neurons to discriminate between learned and not-learned combinations.

To summarize, although synaptic connectivity is random, IS learning causes some dendrites to specialize and respond preferentially to certain combinations of stimuli. Dendrites can then perform a first stage of non-linear integration that is amplified for learned combinations of inputs. This will allow neurons to discriminate learned from not-learned combinations while processing contexts and stimuli at the network level.

### Neuron and network processing of XOR-like combinations

Here we investigate the behavior of a biophysically realistic model of the cerebral cortex during real time recall of a response when presented with a combination of context and stimulus. In particular, we study to what extent processing of XOR-like combination depends on the synaptic matrix generated by IS learning. To this aim, the different types of dendrites are randomly attributed to the neurons of the network, according to their calculated probabilities (Equation 2 and 3). Given that neurons have far fewer dendrites than the total number of types of dendrites, different neurons have different sets of dendrites. However, those neurons are randomly attributed to the populations coding for the contexts, stimuli, and responses. The different populations of neurons have therefore very similar distributions of the types of neurons (defined by their types of dendrites). The fact that different populations have the same types of neurons makes these populations unable to discriminate *a priori* between combinations, because, on average, their neurons respond similarly to the different combinations. This leaves the discrimination between precise combinations to IS learning that shapes the values of potentiation within dendrites as a function of what the post-synaptic neuron codes for (i.e., to which population it belongs). IS learning amplifies synaptic efficacy between neurons activated in combination during learning, compared to efficacy between neurons not activated in combination. A result of IS learning is that, within each population, some neurons have a distribution of dendrites that have learned certain combinations better than others. At the network level, the synaptic matrix is filled with the values of synaptic efficacy calculated according to the IS learning algorithm applied to each type of dendrite according to the learning protocol of the rule (Equation 15).

After IS learning, and non-linear amplification of synaptic inputs, dendritic currents transmitted to each neuron are larger after synaptic inputs from learned combinations than after inputs from not learned combinations. A consequence is that neurons that have a subset of dendrites responsive to a learned combination will respond to this combination (or to several combinations if they have different subsets of dendrites responsive to different combinations). At the level of the populations of neurons, each population has subsets of neurons responsive to the different learned combinations of items. Those subsets of neurons being strongly associated to other neurons of the population (through *J_1_*), they contribute to the activation of the population in response to the combination of items.

**Figure 4:**
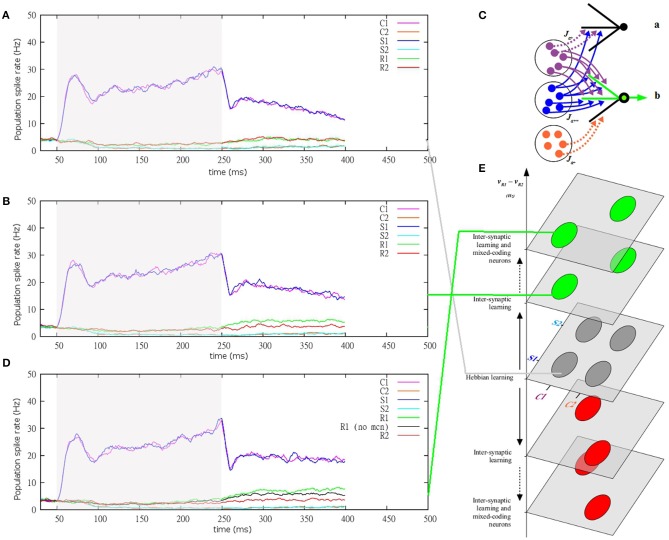
**Attractor states and behavior of the cortical network model selecting a response according to a learned combination C1S1R1 and to the following protocol: neurons at spontaneous activity in absence of selective input for 50 ms; presentation of Context 1 and Stimulus 1 for 200 ms and delay period in absence of selective input for 150 ms). (A–C)** Average spike rate of populations of neurons (averaged over ten trials) coding for the contexts, stimuli and responses (same color as in Figure 1). **(A)** Case of classic Hebbian learning: neurons coding for R1 and neurons coding for R2 both exhibit prospective activity and are not discriminated. **(B)** Case of inter-synaptic learning: neurons coding for R1 exhibit higher prospective activity than neurons coding for R2. **(C)** Example of connectivity to two post-synaptic excitatory neurons that are not selective to any single stimulus or response (NSI-coding neurons) and that do not respond (*a*) or respond (*b*) to combinations of context and stimulus: *a*, receiving only two synaptic inputs from neurons coding for C1 (purple) and S1 (blue) within one of its dendrites (black line), is not activated when the C1S1 combination is presented; *b*, receiving three synaptic inputs from each group of neurons (C1 and S1), each within two dendrites (green lines), is activated when the C1S1 combination is presented. During learning of a rewarded combination (e.g., C1S1R1), in the pool of NSI-coding neurons, some neurons are not activated by the combination (*a*) while others are activated (*b*, mixed-coding neurons). **(D)** Case of inter-synaptic learning with mixed-coding neurons: The contrast between prospective activities of neurons coding for R1 and R2 is larger than in case of inter-synaptic learning alone (rate of neurons coding for R1 with inter-synaptic learning alone **(B)** are reported for clarity, black curve). **(E)** Geometrical representation of the non-linear separability of responses in the space of contexts and stimuli for the XOR-like rule. The axes indicate the type of context (C1 or C2, x-axe) and of stimulus (S1 or S2, y-axe) presented to the network. The z-axe indicate the difference between average spike rates of neurons coding for R1 and for R2 (contrast of prospective activities) during the delay following the presentation of a context and a stimulus. After Hebbian learning, gray circles indicate a null contrast (see **A** connected by a gray line). After IS learning, the contrast is expanded up (green circles) and down (red circles) the z-axe (full black arrows; see **B** connected by a green line). After inter-synaptic learning with mixed-coding neurons, the contrast is further expanded up and down the z-axe (dotted black arrows; see **D** connected by a green line).

The behavior of the network is tested according to a priming protocol very similar to those used in experiments in human and non-human primates, used to probe the dynamics of activation of targets when processing combinations of primes (Balota and Paul, 1996; see Lavigne et al., 2011 for a review). Like in many cortical network models, the current model describes neural spiking dynamics in realistic biophysics terms, with populations of neurons coding for items in memory (Amit et al., 1994; Brunel, 1996; Amit and Brunel, 1997; Pouget et al., 2000; Mongillo et al., 2003; Curti et al., 2004; Romani et al., 2006). The types of activities of the populations of neurons are explained by reverberating activation between excitatory neurons connected with potentiated synapses (Amit et al., 1994; Amit and Brunel, 1997). Here, during a trial, the presentation of the context and stimulus corresponds to an external input to the corresponding populations of neurons, which exhibit elevated spike rates (strong perceptive response). After the context and stimulus offset, the corresponding neurons exhibit retrospective persistent activity that remains stronger than spontaneous activity. This behavior reproduces the elevated firing rates of neurons following the presentation of the stimulus they code for, as reported in non-human primates (Fuster and Alexander, 1971; Miyashita, 1988; Miyashita and Chang, 1988). Such retrospective stimulus-specific activity is regarded as the activation of items in working memory following their presentation (Amit and Brunel, 1997; Brunel and Wang, 2001; Haarmann and Usher, 2001; Renart et al., 2001; Amit et al., 2003). Following the presentation of the context and stimulus, neurons of the corresponding populations are sufficiently activated to activate in turn neurons coding for different but associated items. This behavior reproduces the increasing firing rates of neurons coding for associates to the stimulus presented before their actual presentation (prospective activity), also as reported in non-human primates (Miyashita, 1988; Miyashita and Chang, 1988; Sakai and Miyashita, 1991; Erickson and Desimone, 1999; Rainer et al., 1999; Tomita et al., 1999; Naya et al., 2001, 2003a,b; Yoshida et al., 2003; see Fuster, 2001). Such prospective activity, which takes place above the level of spontaneous activity, is regarded as the recall of knowledge according to the stimuli presented (Brunel, 1996; Lavigne and Denis, 2001, 2002; Mongillo et al., 2003; Lavigne, 2004; Lavigne and Darmon, 2008). Here, prospective activity is generated not by a single stimulus but rather by a combination of a context and a stimulus (Figure 4; Fusi et al., 2007; Rigotti et al., 2010a,b; Lavigne et al., 2011, 2012, 2013).

Performing the XOR-like rule requires the network to activate the population of neurons that code for the response learned in combination with the presented context and stimulus according to the XOR-like rule (e.g., R1 for C1 and S1). After presentation of C1 and S1, the activation of R1 is expected to be larger than the activation of the alternate response R2. We therefore compare the levels of prospective activities of neurons coding for the two responses after presentation of a given combination of context and stimulus (here C1S1). The performance of the network is tested after Hebbian learning, after IS learning, and after IS learning with mixed-coding neurons.

In the case of Hebbian learning, the presentation of the pair of context and stimulus triggers a perceptive response followed by retrospective activity of the neurons coding for them. Due to the Hebbian synaptic matrix, retrospective activity of the context and stimulus leads to the prospective activity of the two responses (Figure 4A). However, the network does not discriminate between the two responses that are activated at the same level, failing to perform the rule. This is because Hebbian learning generates a homogeneous distribution in the values of synapse potentiation between any pair of neurons coding for a context, a stimulus and a response, regardless of the dendrite considered (Figure 2I).

In the case of IS learning, the population coding for the expected response—learned in combination with the context and stimulus—exhibits a higher level of prospective activity than the population coding for the other response (Figure 4B). The IS learning algorithm generates dendrite-specific values of synaptic efficacy, which in turn contribute to the different amounts of dendritic currents that correspond to the different combinations, depending on the response the post-synaptic neuron codes for. This allows the network to perform the rule by discriminating between the two responses in accordance with the combination of context and stimulus.

To summarize, the geometrical representation of the XOR problem shows that, in the case of Hebbian learning, the difference between the prospective activities of neurons coding for the two possible responses (R1-R2) is null in the plane describing the space of contexts and stimuli (Figure 4E). IS learning extends the space of responses to the third dimension, where the activities of neurons coding for R1 and R2 become differentiated.

### Synergistic effects of is learning and of mixed-coding neurons

The computing of XOR-like combinations has been shown to be possible in connectionist models thanks to additional neurons that are organized within a hidden layer and that perform non-linear integration of the inputs before the intervention of the output neurons (e.g., Rumelhart and McClelland, 1986). Along these lines, recent experiments have reported that the prefrontal cortex includes large numbers of neurons that code for abstract combinations of stimuli and responses (Bongard and Nieder, 2010; Rigotti et al., 2013). Computational modeling has shown that these mixed-coding neurons allow XOR-like combinations to be performed at the network level, in a similar way to the function of the hidden layer (Rigotti et al., 2010a,b, 2013; Bourjaily and Miller, 2011a,b, 2012).

In the present model, on the one hand, coding neurons are attributed randomly to the contexts, stimuli or responses (e.g., R1), on the basis of their relation to single stimuli or response. Coding neurons are those that receive inputs (*I_sel_*) and fire whenever the single stimulus or response is present. They are not selected as a function of any *a priori* responsiveness to particular combinations of several items (e.g., C1S1). On the other hand, non-single-item (NSI)-coding neurons do not respond to any single context, stimulus, or response. Different NSI-coding neurons have different types of dendrites that make some of them responsive to some combinations of context and stimulus. For example, a neuron receiving three synaptic inputs from each group of neurons coding for C1 and S1 would be activated by the combination of C1 and S1 (neuron *b* of Figure 4C). This mixed-coding neuron would then be activated during a rewarded trial (e.g., C1S1R1). From this starting point, the IS learning algorithm considers its up-state and potentiates synapses connecting this mixed-coding neuron to neurons coding for C1, for S1 and for R1. Mixed-coding neurons are the object of IS learning in the same way as neurons that are activated directly in relation to the rewarded response R1 (green) (other mixed-coding neurons are activated by the combinations C1R1 and S1R1 while learning the C1S1R1 combination). As a result, the synapses between mixed-coding neurons that respond to the combination C1S1 and neurons coding for R1 are strongly potentiated (Figure 4C).

After IS learning, all mixed-coding neurons that respond to the C1S1 have potentiated synapses with C1 and S1. As a consequence, when C1 and S1 are presented to the network, the populations of neurons coding for C1 and S1 will exhibit retrospective activity, and associated mixed-coding neurons will exhibit prospective activity (Figure 4D). Given that those mixed-coding neurons also have potentiated synapses with neurons coding for R1 (through *J_1_*), their prospective activity will intensify prospective activity of the neurons coding for R1. They behave as if they increase the size of the population R1 and magnify its response to the C1S1 combination. More precisely, only mixed-coding neurons that where responsive to the C1S1 combination prior to learning have, after learning, potentiated synapses with neurons coding for R1. After learning, those mixed-coding neurons that help intensify the activity a population of coding neurons (R1) are the ones that better respond to the related combination C1S1. As a result, mixed-coding neurons behave in synergy with neurons coding for R1 and increase the selectivity of the population R1 to combinations that are effectively learned (e.g., C1S1).

More generally, during learning of the C1S1R1 combination, a fraction of NSI-coding neurons receive synaptic inputs by combinations of C1 and S1, C1 and R1, S1 and R1, or, less frequently, C1, S1, and R1. Considering here the most frequent and simplest cases of mixed-coding neurons that are responsive to combinations of two items (C1S1, C1R1, or S1R1, respectively), IS learning creates populations of mixed-coding neurons that have synapses potentiated with neurons coding for C1, S1, and R1. The same mechanism happens during learning of the C1S2R2, C2S1R2, and C2S2R1 combinations.

The effect of mixed-coding neurons was tested using the same network architecture as in Figure 1D, with 2.5% of the NSI-coding neurons responsive to learned combinations (mixed-coding neurons) selected for IS learning. After learning, their activity during processing of (e.g.,) C1S1 combination was measured as the one of the population coding for R1 (mixed-coding neurons contributed to 10% of the size of the population of neurons in prospective activity in response to C1S1. To compare *ceteris paribus* with the case of IS learning without mixed-coding neurons (B), the populations size is kept constant at 400 neurons. Results show that mixed-coding neurons increase the level of prospective activity of the population coding for the response R1 after presentation of the context C1 and stimulus S1, according to the learned combination C1S1R1 (Figure 4C). The discrimination between responses R1 and R2 is improved compared to IS learning alone (see Figure 4E for a geometrical representation). Results show that the combined effects of IS learning and of mixed-coding neurons improve the network performance in the processing of XOR-like combinations.

## Discussion

The present study proposes an inter-synaptic learning algorithm of XOR-like combinations in randomly connected networks. IS learning takes into account recent experimental evidence on synaptic potentiation of groups of synapses at the level of dendritic branches (Govindarajan et al., 2011; see Iannella et al., 2010; Fu et al., 2012). The IS learning algorithm formalizes the inter-synaptic amplification of potentiation and of depression of synapses as a function of the other synapses that are co-active or not within a dendrite. It causes some dendrites to specialize to respond preferentially to some learned combinations of inputs than to others.

### Synergistic effects of is learning and non-linear dendritic integration

The performance of the network relies on a synergy between non-linear integration of functionally linked synaptic inputs within dendritic branches, and inter-synaptic learning within these same branches that amplify potentiation (or depression) of groups of synapses with correlated (or uncorrelated) activity. The synergy between processing and learning is reciprocal: IS amplification of potentiation or depression depends on the number of co-active synapses in each dendrite, and non-linear dendritic integration is proportional to the number of co-active synapses and to their efficacy. The mathematical formalism of these joint mechanisms shows that IS learning of combinations according to a XOR-like rule does not requires network pre-wiring. Instead, effective learning is possible based on totally random distributions (1) of the synapses in the different dendrites of the dendritic arbor, (2) of the resulting types of dendrites in the different neurons, and (3) of the neurons in the different populations coding for the items. IS learning therefore depends exclusively on the correlational structure of the rewarded combinations of inputs and responses. It amplifies the potentiation or depression of synapses only as a function of the learned combinations of context and stimulus at the level of the individual dendrites. This in turn makes dendrites able to respond more strongly to certain specific combinations of synaptic inputs. We have shown that non-linear dendritic integration is necessary for IS learning to be efficient on the dendritic response. We have described the simplest case of linear IS, with both probabilities of potentiation *a_ij_*(*D*) (Equation 7) and of depression *b_ij_*(*D*) (Equation 10) depending linearly on the number of synapses *n*. Non-linear IS learning could amplify even further the efficacy of synapses that are co-active in the same dendrite or in different dendrites.

The combination of IS learning at the level of dendrites and of the fact that neurons have only a subset of the possible types of dendrites (here 50 dendrites over several millions of different types of dendrites) result in neurons that are more responsive to particular combinations of items than to others. The learned specialization of neurons makes them able to respond preferentially to some of the learned combinations. This allows the network, when presented with a combination of context and stimulus, to select the response that is appropriate for the XOR-like rule. Once the combinations have been learned, the response selection relies solely on the combination processed.

### Non-local is learning and global processing of combinations

The IS learning rule dictates how potentiation and depression of synapses is amplified as a function of the activity of the pre- and post-synaptic neurons, and also as a function of the activity of other pre-synaptic neurons within the dendrite considered. IS learning generates variable levels of synaptic potentiation depending on the type of dendrite considered. Different values of potentiation can exist between pre- and post-synaptic neurons from two given populations, even though the pre-synaptic neurons all come from the same population and the post-synaptic neurons also come from another population. With respect to a given post-synaptic neuron, the efficacy of synapses—from pre-synaptic neurons that code for other items—depends on the type of dendrite considered. This is due to the fact that IS learning depends on all of the synapses grouped within the same dendrite. The IS learning algorithm updates synaptic efficacy by amplifying potentiation or depression of each given synapse as a function of the activity of its pre- and post-synaptic neurons and also of other pre-synaptic neurons having contact within the same dendrite. On this basis, the IS learning rule is non-local and optimizes a given synapse efficacy as a function of the activity of other synapses with several other neurons coding for different items, depending on the combinations of these items.

The performance of the network in processing XOR-like combination rules relies on its ability to activate a given item not only as a function of another item but as a function of the combination of other items. After random connectivity and IS learning within dendrites, each population of neurons coding for a given item comprises sub-groups of neurons that better respond to particular combinations of inputs than to others. When presenting a context and a stimulus to the network, the corresponding populations exhibit a perceptive response followed by retrospective activity. This triggers prospective activity of the population coding for the response learned within the same combination, at a higher level than the one of the other response. Due to IS learning, different states are reached depending on the combinations of context and stimulus presented, that differ in the level of prospective activity of the two responses (Figure 4B). This is not the case with Hebbian learning that generates states that do not differ in terms of the level of prospective activity of the responses (Figure 4A). The prospective activation of a given response is based on the global pattern of retrospective activity of a context and a stimulus that are combined together.

### Synergistic effects of is learning and mixed-coding neurons

Processing of XOR-like combinations has been reported to benefit from the existence of mixed-coding neurons (Rigotti et al., 2010a,b, 2013; Bourjaily and Miller, 2011a,b, 2012). In the model proposed here, the addition of mixed-coding neurons to IS learning increases the contrast between the adequate and inadequate responses to a given combination of context and stimulus. IS learning and mixed-coding neurons act in synergy to enhance the performance of the network. First, IS learning applies to those mixed-coding neurons that better respond to combinations of items effectively learned, and not to mixed-coding neurons that respond to combinations not learned. Second, mixed-coding neurons that have potentiated their synapses with neurons coding for combined context, stimulus, and response (e.g., C1S1R1) intensify the prospective activity of the adequate response R1 and enhance the contrast between prospective activities of R1 and R1. Those mixed-coding neurons therefore contribute to increase the selectivity of the network to combinations of context and stimulus.

IS learning could also reduce the number of mixed-coding neurons that are necessary to perform a given rule, and enhance the ability of mixed-coding neurons to perform more complex rules. In the current study, the most frequent cases of mixed-coding neurons responsive to combinations of C1S1, C1R1, or S1R1 have been considered. The mixed-coding neurons activated by one of these combinations have been considered as neurons coding for R1, S1, or C1, respectively. An additional category of NSI-coding neurons can also respond to combinations of three items (Context-Stimulus-Response: e.g., C1S1R1). Those neurons would not behave like those coding for items, but would form additional populations coding for specific combinations of three items. Such mixed-coding neurons have dendrites with a maximum number of synapses from neurons from three different populations: four synapses from C1, four from S1, and four from R1 (instead of, e.g., four from C1 and four from S1). Such neurons are rare and the importance of their role in enhancing the performance of the network will be considered in a further study.

### Generalization to context-dependent combinatorial processing

Behavioral responses to a given stimulus can vary depending on the processing of other stimuli, motivations and goals (Drea and Wallen, 1999; Platt and Glimcher, 1999; Handel and Glimcher, 2000; Wise and Murray, 2000; Wallis et al., 2001; Hobin et al., 2003; see Salinas, 2004; Muhammad et al., 2006). The activation of items in working memory generating responses can be experimentally measured in humans with semantic priming protocols. Priming effects correspond to response times for processing target words that are shorter when targets are associated with preceding prime words than when they are not (Meyer et al., 1972; Schvaneveldt and Meyer, 1973; see Neely, 1991; Hutchison, 2003). The resulting priming effect is assumed to measure recall as a function of the level of activation of the target by the preceding prime, which depends in turn on the strength of the prime–target association (Abernethy and Coney, 1993; Coney, 2002; Hutchinson et al., 2003; Frishkoff, 2007; see Chiarello et al., 2003) estimated prior to testing (McRae et al., 1997; Cree and McRae, 2003; see Nelson et al., 1999).

Recent studies have suggested that priming effects are not systematic, but rather depend on the specific tasks given to participants, as they presumably activate contextual representations that orient the processing of information (Bermeitinger et al., 2008, 2011; Kiefer and Martens, 2010; see Gollwitzer and Kinney, 1989; Kiefer, 2007; Rothermund et al., 2008; Martens and Kiefer, 2009; Spruyt et al., 2009). Multiple priming experiments show that the activation of a target by a prime depends on another contextual prime, depending on their association with this target (McNamara, 1992; Balota and Paul, 1996), on the primes-target delays (Lavigne and Vitu, 1997; Lavigne et al., 2011) and on the strength of the association between each prime and the target (Lavigne et al., 2012, 2013; see Lavigne et al., 2011 for a cortical network model). In particular, the target is activated in a context of two associated primes, whereas it is not activated if very weakly associated (Lavigne et al., 2011, 2012, 2013) or almost not associated (Khalkhali et al., 2012) with the primes. Such contextually dependent priming effects, visible when the primes are weakly associated to the target, could correspond to supra-threshold activation of the target under the condition that two primes are presented (see Lavigne et al., 2011 for a model). In this case, a simple non-linear integration of the activations generated by the two primes can be effective to activate the target, while a single prime is not sufficient to activate the target. In that case, supra-threshold activation of the target by the two primes can arise whatever the primes considered as long as they are associated with the target. In other words, the activation of a target does not depend on any particular combination of specific primes, but rather on the cumulative activation whatever the primes involved. However, complex contextual rules dictate that a target will only be activated by precise combinations of specific primes, and that other prime combinations will not activate the target even though they are each individually associated with it.

Though context-dependent activation does not necessarily involve XOR-like combinations, the IS learning algorithm could improve the performance of the network in discriminating between a particular response to a stimulus in a given context from a set of responses that are also associated with this stimulus but in different contexts. In the priming protocol simulated here with the context and stimulus as primes, IS learning generates stable states of the network that are representative of the learned combinations, by discriminating the level of prospective activity of the responses in different states. A consequence is that, after presentation of a context and a stimulus, the evolution of the state of the network indicates that the context selects which response is more activated by the stimulus. The context selects a subset of possible trajectories within the attractor landscape describing the different possible responses that can be activated by the stimulus. Contextual processing would then correspond to the selection of some trajectories within the attractor landscape. More generally, the IS learning algorithm provides us with a computational framework to describe how every context or stimulus can be a selector of which dynamics can exist and which cannot. An interesting insight is that a context would not only activate a response in addition to the stimulus, but would enable a path through which a stimulus can activate a response.

### Conflict of interest statement

The authors declare that the research was conducted in the absence of any commercial or financial relationships that could be construed as a potential conflict of interest.
